# MUC1 as a Survival Effector of Radiotherapy‐Induced Epithelial Hybrid States in Basal‐Like Breast Cancer

**DOI:** 10.1002/ijc.70487

**Published:** 2026-04-18

**Authors:** Garyfallia Pantelaiou‐Prokaki, Husam Bamahmoud, Nadine S. Georges, Shaishavi Jansari, Annika Jung, Daniela Grimm, Evangelos Prokakis, Fabian Alexander Gayer, Maryam Deldar, Christian Dullin, Julia Gallwas, Florian Wegwitz, Frauke Alves

**Affiliations:** ^1^ Translational Molecular Imaging Max Planck Institute for Multidisciplinary Sciences Göttingen Germany; ^2^ Department of Gynecology and Obstetrics University Medical Center Göttingen Göttingen Germany; ^3^ Department of Urology Medical Faculty and University Hospital Düsseldorf, Heinrich Heine University Düsseldorf Düsseldorf Germany; ^4^ Department of Urology University Medical Centre Göttingen Göttingen Germany; ^5^ Institute for Diagnostic and Interventional Radiology, University Medical Center Göttingen Göttingen Germany; ^6^ Department of Diagnostic and Interventional Radiology University Hospital Heidelberg Heidelberg Germany; ^7^ Clinic for Hematology and Medical Oncology University Medical Center Göttingen Göttingen Germany

**Keywords:** basal‐like breast cancer, epithelial/mesenchymal hybrid state, irradiation, MUC1, syngeneic mouse model

## Abstract

Basal‐like breast cancer (BLBC) is characterized as the most aggressive and poorly understood breast cancer (BC) subtype. Next to surgery, conventional chemotherapy and immunotherapy, radiotherapy (RT) represents one of the primary treatment options. Transcriptional plasticity is largely responsible for BLBC's ability to rapidly adapt to genotoxic stress, enabling cell survival, tumor repopulation, and disease progression. Our research group has recently shown that chemotherapy primarily elicits the epithelial‐to‐mesenchymal transition (EMT) transcriptomic program by epigenetically derepressing the expression of several important EMT‐promoting factors, which are crucial for drug tolerance and disease relapse. However, the induced transcriptomic programs upon RT in BLBC have been elusively addressed. To fill this gap, we leveraged high‐throughput bulk and spatial transcriptomic data in a syngeneic mouse model of BLBC, corroborated by publicly available patient‐derived bulk transcriptomic data and combined with an in vitro 3D BLBC model approach. Contrary to our expectations, we found that irradiated basal‐like breast cancer (BLBC) tumors shifted their epithelial/mesenchymal hybrid state toward a stronger epithelial gene expression program, ultimately driving MUC1 (Mucin 1) expression as a key mediator of cell survival following RT. Altogether, our study uncovers a new aspect of the BLBC transcriptional plasticity elicited by RT, leading to a distinct hybrid phenotypic state from that upon chemotherapy. Concluding, these data underscore the potential clinical value of MUC1 as a therapeutic target or prognostic marker to optimize BLBC patients’ response to RT.

## Abstract (highlights)

What's new?

Basal‐like breast cancer (BLBC) is aggressive and often exhibits inherent or acquired resistance to radiotherapy (RT), a primary treatment option. RT resistance is underpinned by molecular alterations, many of which are poorly understood. Here, RT‐induced transcriptomic changes were analyzed using bulk and spatial sequencing in a BLBC mouse model and validated with patient data and *in vitro* 3D models. RT was found to stimulate an epithelial/mesenchymal hybrid state, with increased epithelial character and *MUC1* induction. *MUC1* expression mediated cell survival following RT. The observed response differs from chemotherapy‐induced epithelial‐to‐mesenchymal transition programs, revealing a distinct RT transcriptional adaptation in BLBC.

## Graphical abstract

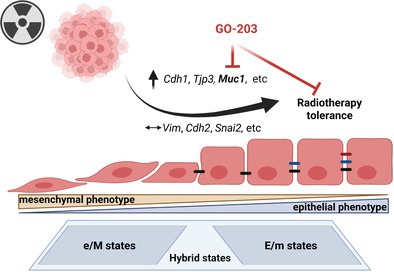

**Abbreviations**
BC: breast cancer
BLBC: basal‐like breast cancer
CAF: cyclophosphamide, adriamycin, 5‐fluorouracil
EMT: epithelial‐to‐mesenchymal transition
GSEA: gene set enrichment analysis
IHC: immunohistochemistry
MUC1: mucin‐1
NGS: next‐generation sequencing
OCT: optimal cutting temperature
RT: radiotherapy
TNBC: triple‐negative breast cancer
WAP‐T: whey acidic protein‐T antigen

## 1 Introduction

Breast cancer (BC) is the most common cancer type with one of the highest mortality rates in the female population worldwide [1]. BC is a heterogeneous disease and is classified into four major subtypes based on the expression of the hormone receptors of estrogen (ER) and progesterone (PR), and the human epidermal growth factor receptor 2 (HER2) [2, 3]. With the advent of targeted therapies against these receptors, there has been considerable improvement of the patients' survival outcomes, particularly in luminal A (ER^+^/PR^+^/Ki67^low^), luminal B (ER^+^/PR^low/−^/Ki67^high^, which sometimes express HER2) and HER2‐enriched (ER^−^/PR^−^/HER2^+^) patients [3]. However, triple‐negative (ER^−^/PR^−^/HER2^−^) BC (TNBC) patients, a subtype largely overlapping with the basal‐like BC (BLBC) molecular subtype (10%–15% of all BC cases), do not benefit from such therapies [3]. While immune checkpoint inhibitors (ICIs) have shown potential in TNBC treatment, particularly in combination with chemotherapies, their overall efficiency remains modest [4]. In addition, 40% of BC patients who receive radiotherapy (RT) are surviving, but disease recurrence and poor survival outcomes may still occur, depending on the diagnosed stage of BC lesions [5]. Parameters such as the locoregional state of the lesion, the hormone receptor status, and the patient's age are taken into consideration for treating BC patients with RT. In the case of BLBC, increased risk of locoregional recurrence, disease dissemination, and heightened mortality upon RT is quite common [6]. Several ongoing studies aim to pinpoint the threshold at which BLBC lesions survive RT and to identify therapeutic targets that enhance RT sensitivity. However, progress has been limited due to the dose‐dependent systemic toxicities and off‐target effects associated with agents targeting newly identified drivers of RT resistance [7]. Therefore, it remains a critical need to develop safer and more effective strategies for overcoming RT resistance in BLBC.

In the current study, we aimed to investigate the molecular factors that enable escape mechanisms for BLBC to survive RT. For this, we utilized the well‐established orthotopic immunocompetent WAP‐T mouse model, which has been widely used in our previous studies, reliably mimicking BLBC, the syngeneic BLBC cell line H8N8 [8] for several functional assays as well as bulk and spatial transcriptomic approaches [9, 10, 11, 12, 13]. Additionally, we leveraged publicly available patient‐derived bulk transcriptomic data. Surprisingly, we found that RT shifted the epithelial/mesenchymal hybrid state (hybrid state) of the tumor cells toward a stronger epithelial gene expression program in vivo and in vitro via the upregulation of several epithelial markers. In line, we identified the epithelial marker *Muc1* being implicated in increased BLBC cell resistance to RT, while gene silencing or its pharmacological interference further potentiated the cytotoxic effects of RT in BLBC. Therefore, our study reveals a previously unknown role of the RT‐induced epithelial phenotype in contributing to the aggressive nature of BLBC and positions MUC1 as a novel pharmacological target to enhance patients' responses to RT‐based therapies.

## 2 Materials and Methods

### 2.1 Cell Lines, Cell Culture

The murine H8N8 cell line was established from a WAP‐T primary mammary tumor, as previously described in our published work [8]. mH8N8 (mesenchymal) and eH8N8 (epithelial) cells were maintained in high glucose (4.5 g/L D‐glucose) Dulbecco's modified Eagle's medium (DMEM), containing 10% fetal calf serum (FCS, both from Thermo Fisher Scientific Corp.) at 37°C in a humidified atmosphere with 5% CO_2_. For the irradiation of tumor spheroids, the x‐ray radiation device KUBTEC XCELL 225 was employed using a total dose of 0, 1, 2, 4, or 5 Gy, based on the manufacturer's instructions. For further details on cell culture methods and assays, please refer to the Supporting Information. All experiments were performed with mycoplasma‐free cells.

### 2.2 Orthotopic WAP‐T Mouse Model and Irradiation

**FIGURE 1:**
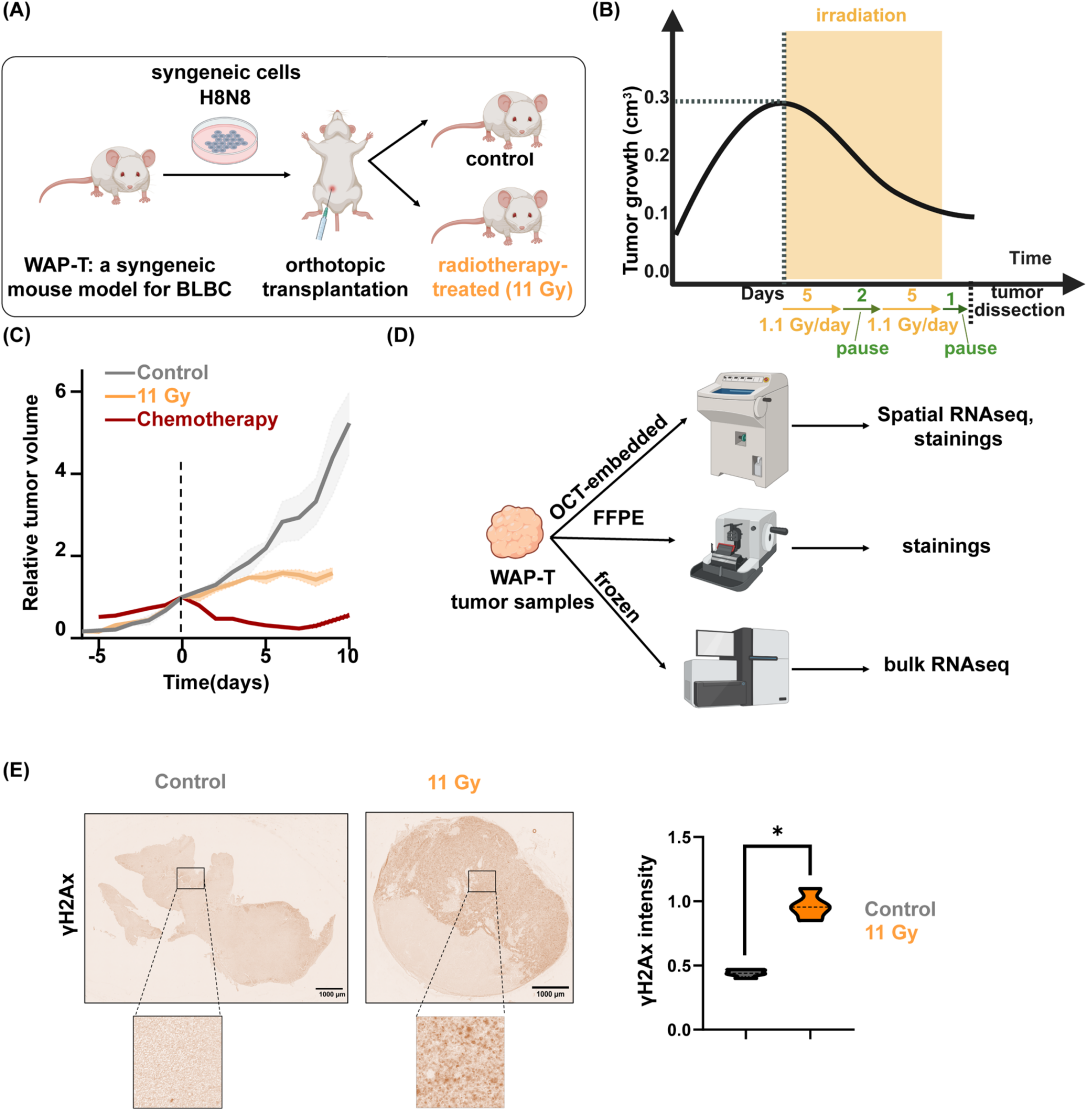
WAP‐T mice respond to RT showing typical signs of DNA damage. (A) Schematic representation of the animal experimental procedures: H8N8 tumor cell cultivation, transplantation in female non‐induced transgenic heterozygous WAP‐T mice, and irradiation. (B) Groups of control and 11 Gy‐treated tumor‐bearing mice are depicted once tumors reached 0.3 cm^3^ volume. (C) Tumor growth kinetics of control, irradiation‐ (total dose of 11 Gy) or CAF‐ (cyclophosphamide, adriamycin, 5‐fluouracil, results taken from previous published study [13]) treated H8N8 tumors are shown. Tumor volumes were normalized to the tumor volume at the start of therapy (dashed horizontal line, day 0). Tumor volumes were measured in situ on live mice by caliper measurements (Control *n* = 5, irradiated *n* = 4, and CAF *n* = 12). (D) Schematic representation of the experimental workflow following the tumor dissection at the end of the tumor growth kinetic experiment. (E) Immunohistochemical detection of γH2Ax in control‐ and irradiated‐tumors (left panel) and staining quantification (right panel). Black scale bar: 1000 μm. Statistical test: (E) Mann–Whitney test, *p < 0.05. All experiments were performed in biological triplicates.

1 × 10^6^ H8N8 tumor cells suspended in 20 μL PBS were orthotopically implanted into the right abdominal mammary gland of ketamine/xylazine anesthetized (2.5–3 μL/g body weight) syngeneic female WAP‐T‐NP8 mice (Figure 1A), as described previously [14]. Transplanted animals' fitness was assessed, body weight was determined, and tumor volumes were measured using a caliper three times a week for the entire duration of the experiments.

Irradiation treatment was started when a tumor volume of 300 mm^3^ was reached (Figure 1B). Four female mice (body mass 25.8 ± 4.6 g, age at tumor cell implantation 131.4 ± 42.1 days) were irradiated with the XCELL RS225 device using a total dose of 11 Gy (5 days 1.1 Gy per day, 2 resting days, 5 days 1.1 Gy per day). Five tumor bearing mice (body mass 25.7 ± 6.5 g, age at tumor cell implantation 118.9 ± 44.4 days) were not treated and served as control group. Twenty four hours after the last treatment or in case of the untreated mice at the same day, mice were sacrificed using CO_2_ in combination with cervical dislocation. The tumors were dissected and divided into three portions: one fixed in formalin and paraffin‑embedded for histology and immunohistochemistry, one OCT‑embedded for spatial transcriptomics and immunofluorescence, and one snap‑frozen in liquid nitrogen and stored at −80°C for bulk RNA extraction.

### 2.3 Histology and Immunostaining

Paraffin‐embedded tumor tissues were cut into sections of 5 μm and deparaffinized. Immunostaining of γH2Ax and MUC1 was performed as described in Supporting Information. Primary and secondary antibody dilutions as well as individual conjugations used are listed in Table S6.

### 2.4 siRNA Transfection

Cells were reverse transfected with siRNA using Lipofectamine RNAiMAX (Thermofisher), according to the manufacturer's instructions. siGENOME siRNAs (Dharmacon) were acquired at Horizon Discovery Ltd. and utilized as smart pools of four different *Muc1*‐specific siRNAs equimolarly mixed (each 20 μM). For more details, please refer to Supporting Information (Table S1).

### 2.5 RNA Isolation and cDNA Synthesis

For cell culture, RNA was isolated using a NucleoSpin RNA isolation kit (Macherey and Nagel) according to the manufacturer's instructions. For tissue processing and subsequent bulk‑RNA sequencing, we used the Trizol‑chloroform extraction protocol as established in our laboratory. For more details, see Supporting Information.

### 2.6 Real‐Time Quantitative PCR

For quantitative real‐time polymerase chain reaction (RT‐qPCR), samples were quantified by using the method of the standard curve. The sequences of the RT‑qPCR primers, the PCR reaction composition, and the PCR program used are provided in Tables S2–S4 in Supporting Information.

### 2.7 Bulk RNA‐Sequencing Library Construction and Analysis of Raw Data

mRNA was purified using poly‐A isolation beads and converted into cDNA for later library construction using the TruSeq RNA Sample Prep kit v2 (Illumina) according to the manufacturer's instructions. mRNA‐seq data quality was checked using the FastQC Read Quality reports (Galaxy Version 0.72). Sequences were trimmed up to 11 bases from the 5'end with the “Trim trailing and leading characters” tool (Galaxy Version 0.0.1). All sequences were aligned to the mouse genome (mm10) using the RNA STAR gapped‐read mapper for RNAseq data (Galaxy Version 2.5.2b‐2). To quantify the gene sequence fragments (reads) which were allocated to a gene of the reference mouse genome, featureCounts (Galaxy Version 1.6.0.2) was utilized. Differential gene expression analysis and normalization were performed using the Galaxy DESeq2 (2.11.40.6 + galaxy1) application. For Gene Set Enrichment Analyses (GSEA v.4.1.0.), normalized counts per gene filtered of greater than 10 were analyzed with the following settings in the gene set database Hallmarks (v.2022.1): Number of permutations = 1000; Collapse to gene symbols = No_Collapse; Permutation type = gene_set, metric for ranking genes = Signal2Noise; Max size = 6000; Min size = 15; Number of marker = 1000. The remaining parameters were set as default. Differential gene expression data (DeSEQ2 data) were filtered with basemean > 10, pval < 0.05 and log2FC ≥ 0.5 (UP) or ≤ −0.5 (DN). For more details, see Supporting Information. The sequencing coverage and quality statistics for each sample are summarized in Table S7.

### 2.8 10x Visium Spatial Tissue Optimization, Gateway Gene Expression and Spatial Transcriptomic Analyses

To achieve optimal results in the 10x Genomics Gene Expression workflow, the tissue permeabilization time for our H8N8 cryo tumor tissues had to be determined using the Gateway Tissue Optimization Slide (PN 3000394). For this purpose, 10x Genomics' Visium Spatial Tissue Optimization protocol was carried out according to the manufacturer's instructions (PN 1000313). For spatial RNA seq, tissue sections were placed on the Visium Gateway Gene Expression slide V21S08–020 which contains two capture areas (6.5 × 6.5 mm), each with approx five thousand gene expression spots with unique barcodes and primers were required for the capture and priming of poly‐adenylated mRNA to generate a spatial gene expression library, aided by the Gateway Gene Expression Reagent Kit (PN 1000315). To analyze spatial transcriptomic datasets, 10x Genomics Space Ranger v3.0.0 was utilized to create a gene expression matrix including spatial location from FASTQ and image files. The mm10 genome was used for alignment. The resulting spatial‐transcriptomics data were further subjected to downstream analyses using the Seurat package (v5.1.0) in R following their proposed pipeline [15]. First, data normalization was executed using SCTransform. Post‐normalization, principal component analysis (PCA) was conducted, followed by neighborhood identification (FindNeighbors), cluster detection (FindClusters) and finally dimensional reduction using UMAP (uniform manifold approximation and projection), as implemented in the RunUMAP function. The resulting object was used to access gene expression data for comparative analyses. The sequencing coverage and quality statistics for each sample are summarized in Table S8.

### 2.9 Immunofluorescence Staining of H8N8 Tumorspheres and Tumor Tissues

For more details, see Supporting Information.

### 2.10 Protein Isolation and Western Blot

Protein extraction was carried out with the protease‑inhibitor cocktail listed in Table S5, and the Western‑blotting protocol is described in the Supporting Information.

### 2.11 Use of Publicly Available Patient‐Derived Data

**FIGURE 2:**
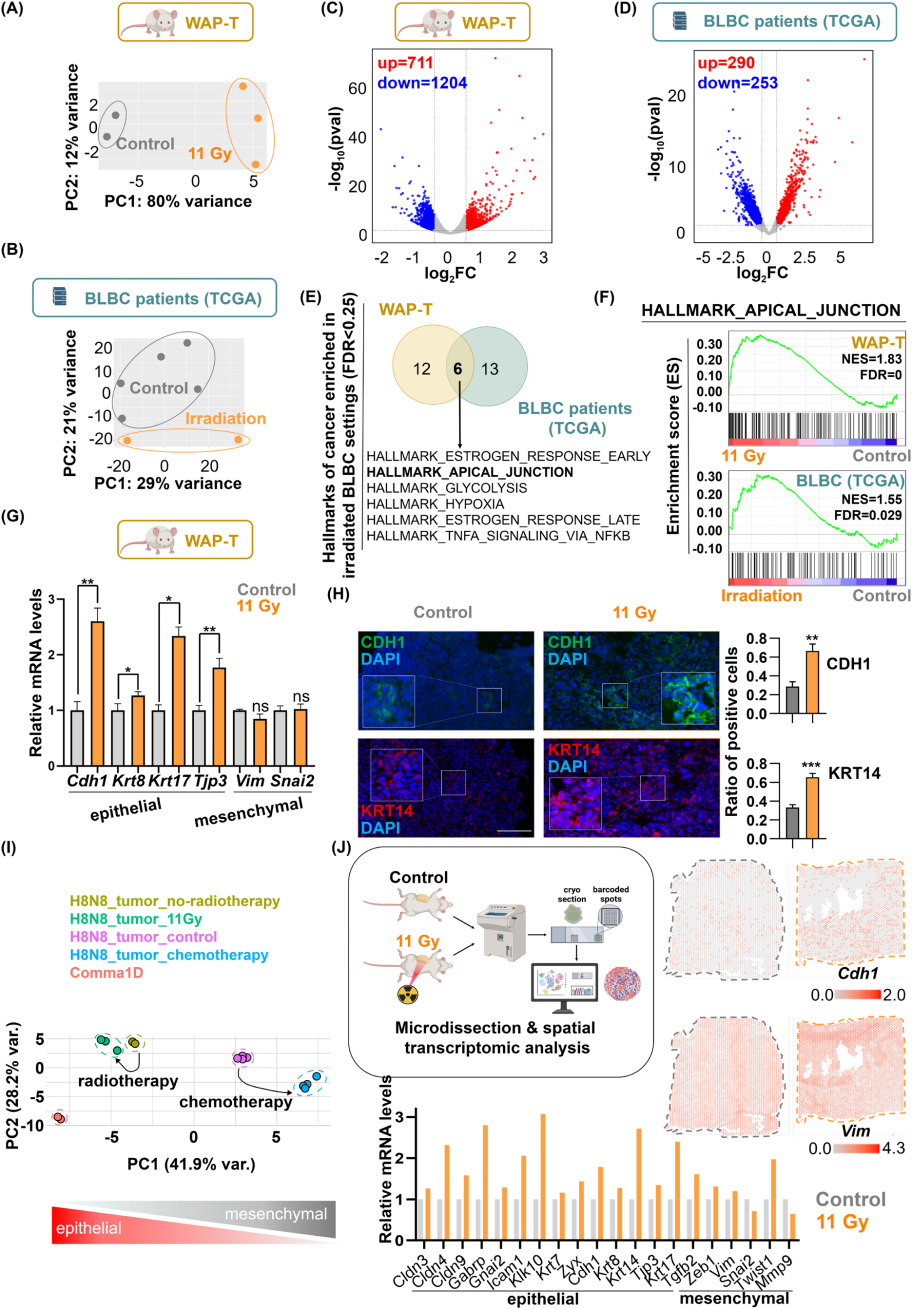
RT elicits the epithelial transcriptomic program in BLBC. (A–D) Principal component analysis (A, B) and volcano plot (C, D) of differentially expressed genes in control‐ and RT‐treated WAP‐T tumors (A, C) and BLBC patients (B, D). (E, F) Venn diagram showing the overlap of gene sets enriched in RT‐treated WAP‐T tumors and patient‐derived BLBC biopsies (E), and the gene set enrichment analysis profile of the gene set HALLMARK_APICAL_JUNCTION in both datasets (F). Source of gene sets: www.gsea‐msigdb.org. FDR, false discovery rate; NES, normalized enrichment score; ES, enrichment score. (G) Real‐time quantitative PCR (RT‐qPCR) of epithelial and mesenchymal genes in control‐ and RT‐treated WAP‐T tumors. (H) Immunofluorescence staining of CDH1 (E‐cadherin) and KRT14 (Keratin 14) in control‐ and RT‐treated WAP‐T tumors (left panel) and the respective quantification of positive cells (right panel). (I) PCA was performed using normalized expression values of a selected panel of epithelial and mesenchymal marker genes (see Figure S1A) across H8N8‐derived samples with chemotherapy [13], control and RT‐treated tumors of this study and Comma1D (SCA1^low^/EPCAM^high^) epithelial reference cells [16] (GEO accession number: GSE182354). Axes correspond to the two leading components obtained from the covariance matrix of normalized gene expression values, illustrating sample variance based on the epithelial and mesenchymal gene set. (J) Schematic workflow of the spatial transcriptomic analysis of cryopreserved control‐ and RT‐treated WAP‐T tumors (upper‐left‐ panel) showing an overall increase of epithelial and modest‐to‐unchanged mesenchymal gene expression in tumors upon RT (lower‐left panel) as in the case of *Cdh1* and *Vim* (see visium slide‐derived spatial heatmap, right panel). Statistical test: (G, H) Student *t*‐test, *p < 0.05, **p ≤ 0.01, ***p ≤ 0.001. All experiments were performed in biological triplicates.

Patient survival data were retrieved from The Cancer Genome Atlas (TCGA, xenabrowser.net) platform to examine the association of *MUC1* expression with patient survival in BLBC patients. The *MUC1* expression cut‐off was selected using the CutoffFinder (v1, https://molpathoheidelberg.shinyapps.io/CutoffFinder_v1/). CNV information was retrieved from cBioportal (www.cbioportal.org) and *MUC1* expression in healthy and BC donors was retrieved using the online tool GEPIA (gepia.cancer‐pku.cn). The therapy history of BLBC patients, whose bulk RNA‐seq datasets have been analyzed in Figure 2, was retrieved from XenaBrowser (xenabrowser.net) and in particular from the BRCA cohort. To identify the BLBC patients analyzed in Figure 2, the following phenotypic variables were selected: (1) “samples”, (2) “radiation_therapy” (NO for Control, YES for irradiated samples), (3) “additional_pharmaceutical_therapy” (NO for both groups) and (4) “additional_radiation_therapy” (NO for both groups) leading to 5 control (TCGA‐B6‐A0I1‐01, TCGA‐A2‐A0T2‐01, TCGA‐B6‐A0I6‐01, TCGA‐B6‐A0WX‐01, TCGA‐E2‐A1LK‐01) and 2 irradiated (TCGA‐A2‐A0YJ‐01, TCGA‐E2‐A1LL‐01) BLBC samples.

### 2.12 Statistical Analysis

Most experimental data were collected from at least three biological replicates *n* = 3. Statistical analyses were performed by GraphPad Prism v8.0.1 (GraphPad Software Inc., CA, USA). Differences between groups were analyzed by the unpaired *t*‐test, Mann–Whitney test, or by one‐way analysis of variance (ANOVA). *p* value < 0.05 was considered to present a significant difference.

## 3 Results

### 3.1 WAP‐T Mice Respond to RT Showing Typical Signs of DNA Damage

In this study, we utilized the WAP‐T mouse model [8], an immunocompetent BC mouse model, well‐established for the study of BLBC to identify the mechanisms sustaining cell survival after RT. Similar to our previous investigations into chemotherapy resistance in BLBC [11, 12, 13], we orthotopically transplanted H8N8 cells into syngeneic WAP‐T recipient mice. Once mammary tumors reached 300 mm^3^, mice were irradiated with 11 Gy total dose (Figure 1A,B), as described in [14]. As expected, irradiation of 11 Gy, as well as the CAF treatment (combination of cyclophosphamide, adriamycin, and 5‐fluorouracil), used in our previous studies, led to tumor shrinkage (Figure 1C) [11, 13]. Dissected tumors 24 h after the final RT dose were either paraffin‐embedded for immunohistochemistry, OCT‐embedded for spatial analyses, or snap‐frozen for bulk transcriptomic analyses. This strategy empowered us to conduct an extensive panel of follow‐up analyses, revealing key biological insights (Figure 1D). As expected, immunohistochemical detection of the phosphorylated form of histone 2AX at serine 139 (γH2AX), which is required for checkpoint‐mediated cell cycle arrest and DNA repair following double‐stranded DNA breaks [17], was significantly higher in RT‐treated BLBC tumors compared to their controls (Figure 1E). In conclusion, BLBC tumors in WAP‐T mice demonstrate the expected response to RT, validating our in vivo BLBC model as adequate for enhancing our understanding of the RT‐resistance mechanisms developed in BLBC.

### 3.2 RT Elicits the Epithelial Transcriptomic Program in BLBC

To get an overview of the transcriptomic programs activated and likely to support tumor cell survival upon RT in BLBC, we first performed bulk mRNA sequencing of RT‐treated versus control H8N8 tumors growing in WAP‐T mice (Figure 2A). In parallel, to improve the translational aspect of our analysis, we retrieved bulk transcriptomic datasets of naïve and solely RT‐treated BLBC patients from the “The Cancer Genome Atlas Breast Cancer” (TCGA‐BRCA) database (Figure 2B). Subsequent differential gene expression analyses identified 711 and 290 upregulated genes in RT‐treated mouse and human tumors, respectively. Additionally, 1204 and 253 genes were found to be downregulated (mouse and human, respectively) (Figure 2C,D). We next performed gene set enrichment analyses (GSEA) on both sets of upregulated genes and compared the enriched datasets (Figure 2E). This approach identified several signaling pathways associated with tumor cell survival following RT in previous reports, validating the relevance of our approach [18, 19, 20, 21]. One specific gene signature, “HALLMARK_APICAL_JUNCTION”, including numerous genes encoding epithelial markers (among others *Cdh1*, *Krt8*, *Krt17*, *Tjp3* and *Muc1*), drew our attention as it was found particularly enriched in BLBC samples surviving RT (Figure 2E,F). This observation was interesting, as our previous investigations, among others, in the same BLBC mouse model identified the opposite EMT process implicated in the resistance to chemotherapies [11, 12, 22]. Moreover, numerous reports have implicated a shift to more mesenchymal phenotypes in RT resistance in various cancers [23]. To validate this striking observation from our analysis finding, we first performed RT‐qPCR and confirmed that all previously mentioned epithelial markers were indeed significantly upregulated in H8N8 tumors upon RT (Figure 2G). We further visualized the upregulation of the epithelial markers CDH1 (E‐cadherin) and KRT14 (Keratin 14) via immunofluorescence staining on cryosections (Figure 2H). Interestingly, additional RT‐qPCR on mesenchymal genes demonstrated no pronounced expression changes, suggesting the induction of a hybrid state with pronounced epithelial character (short E/m‐state) upon RT rather than a full mesenchymal‐epithelial transition (MET) shift (Figures 2G,H and S1A).

We performed principal component analysis (PCA) to assess how RT influences the global epithelial–mesenchymal transcriptional state relative to non‐irradiated controls and chemotherapy‐treated tumors, using a curated panel of epithelial and mesenchymal marker genes. In this analysis, we used the murine normal mammary epithelial cell line Comma1D (SCA1^low^/EPCAM^high^) [16] as a reference for a population with strong epithelial identity. Among the H8N8 tumors, the control, chemotherapy‐treated [13], irradiated [14], and non‐irradiated groups formed distinct clusters, indicating treatment‐dependent transcriptional divergence. Chemotherapy‐treated tumors shifted toward the mesenchymal pole, consistent with the acquisition of mesenchymal‐like features. In contrast, RT‐treated tumors shifted closer to the epithelial reference cluster, supporting the partial re‐establishment of epithelial traits observed in our previous results (Figure 2G,H). Notably, the processing of tumor cells differed between the two experimental settings (RT and chemotherapy), which explains the differences observed in the transcriptomes of the respective control groups. Collectively, these results indicate that, unlike chemotherapy—which promotes hybrid state with pronounced mesenchymal character (e/M‐state) reprogramming—RT induces a transcriptional shift toward a more E/m phenotype within the H8N8 tumor model (Figure 2I).

Consequently, we asked if the induction of this hybrid transcriptional program identified in the bulk mRNA‐seq analyses was due to the emergence of a new tumor cell population with a stronger epithelial phenotype or if it was reflecting the reactivation of epithelial genes tumor‐wide. To answer this question, we performed spatial transcriptomic analysis of control and RT‐treated BLBC tumors using the Visium Spatial Gene Expression platform (10x Genomics) on cryosections. The results demonstrated a strong increase in tumor regions upregulating epithelial markers upon RT (Figure 2J). However, downregulation of mesenchymal markers was not observed, again arguing for the induction of an E/m state (Figures 2J and S1B). In conclusion, RT promotes an E/m state in BLBC, paving the way for further addressing the functional role of this phenomenon in RT resistance in BLBC.

### 3.3 RT Activates the Epithelial Gene Expression Program Leading to an E/m‐ State in BLBC Cells

**FIGURE 3:**
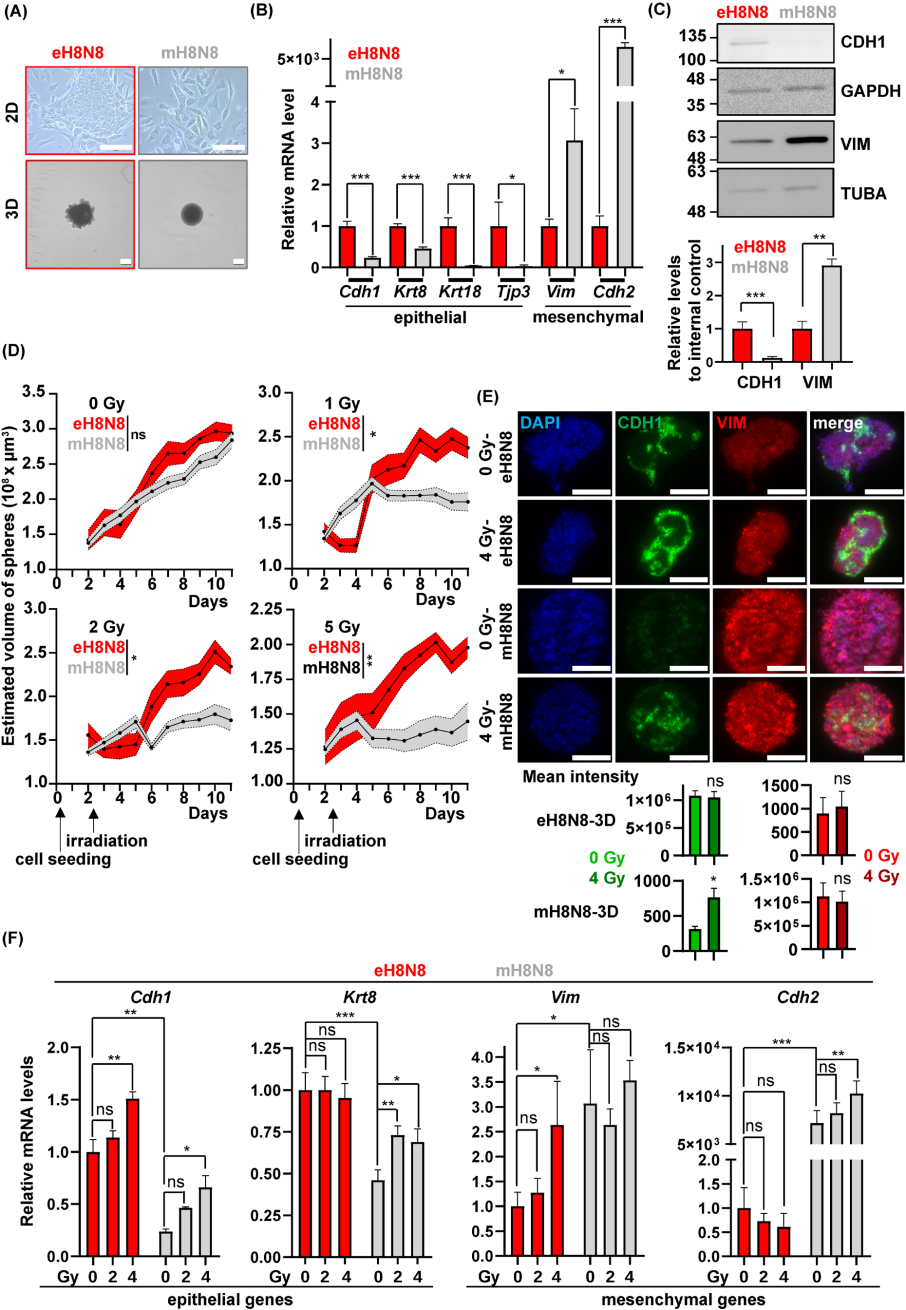
RT activates the epithelial gene expression program leading to an E/m‐state in BLBC cells. (A–C) Representative brightfield microscopy pictures in 2D and 3D culture (A), RT‐qPCR of epithelial and mesenchymal genes (B), and western blotting of CDH1 and VIM with their representative loading controls from one biological replicate (upper panel, C) and the corresponding densitometry (lower panel, C) in eH8N8 and mH8N8 cells are shown. A: White scale bars: 100 μm. (D) Growth kinetics of control, 1 Gy‐, 2 Gy‐ and 5 Gy‐treated eH8N8 and mH8N8 tumorspheres. (E) Immunofluorescence staining of CDH1 and VIM in control and 4 Gy‐treated eH8N8 and mH8N8 tumorspheres (upper panel) and the respective quantifications for each marker (lower panel). White scale bar: 200 μm. (F) RT‐qPCR of epithelial and mesenchymal genes in control, 2 Gy‐ and 4 Gy‐treated eH8N8 and mH8N8 cells. Statistical tests: (B–E) Student *t*‐test (D): Based on the area under the curve, (AUC); (F) One‐way ANOVA. ns: Not significant, *p < 0.05, **p ≤ 0.01, ***p ≤ 0.001. All experiments were performed in biological triplicates.

Building on the discovery of the reactivation of the epithelial transcriptional program within hybrid state tumor cells, we next questioned whether this mechanism was indeed a factor contributing to the emergence of RT resistance. Notably, if such a relationship exists, it could potentially shed light on key therapeutic intervention points and advance our understanding of the interplay between cell phenotype and treatment response. To strengthen our in vivo findings, we took advantage of two variants of the H8N8 cell line that spontaneously emerged while cultivating the cells over time. One of these demonstrated a more pronounced epithelial‐like phenotype (eH8N8; Figure 3A), while the other exhibited a more mesenchymal‐like phenotype in 2D culture (mH8N8; Figure 3A), with each displaying distinct expression levels of specific epithelial markers (*Cdh1*, *Krt8*, *Krt18*, *Tjp3*) and mesenchymal markers (*Vim* and *Cdh2*) on the mRNA (Figure 3B) and the respective protein level (Figure 3C). Moreover, both cell lines show the capacity to grow as 3D tumorspheres under non‐adherent conditions, allowing a closer simulation of the in vivo situation (Figure 3A). Relying on this model, we first tested whether eH8N8 and mH8N8 spheres exhibited different sensitivities to RT treatment. Under normal growth conditions, we detected no significant differences in growth kinetics between the two tumorsphere variants (Figure 3D). However, eH8N8 cells exhibited increased resistance to higher doses of radiation therapy compared to the mH8N8 variant, suggesting that certain aspects of the epithelial program may indeed contribute to cell survival following radiation therapy (Figure 3D). Building on this observation, we next asked if the RT‐induced epithelial‐like gene signature could also be observed in our in vitro setting. As expected, staining of the eH8N8‐ and mH8N8‐derived sphere cryosections after 4 Gy RT showed increased levels of CDH1 (E‐cadherin) and KRT14 (Keratin 14), especially in the mesenchymal cell variant (Figures 3E and S1C). In contrast, levels of VIM remained largely unchanged, consistent with our previous in vivo observations (Figure 2). Subsequent RT‐qPCR analyses on eH8N8 and mH8N8 grown in 2D conditions and treated with different doses of RT revealed an increase of the epithelial markers *Cdh1* and *Krt8*, most notably in mH8N8 cells, but which did not surmount the basal expression levels of eH8N8 cells (Figure 3F). A closer examination of the gene coding for transcription factors (TFs) known to promote the epithelial phenotype and associated with E/m states [24], such as *Ovol1*, *Grhl2*, *Sox9*, and *Cebpd*, revealed consistent upregulation following treatment with higher radiation doses in mH8N8 cells (Figure S2A). Hence, both of our observations in the 2D and 3D RT model systems (B), strongly indicate the activation of the epithelial program sustaining an E/m‐state as a means to better tolerate the cytotoxic effects of RT.

### 3.4 The Expression of the Epithelial Marker MUC1 Is Highly Induced Upon RT in BLBC

**FIGURE 4:**
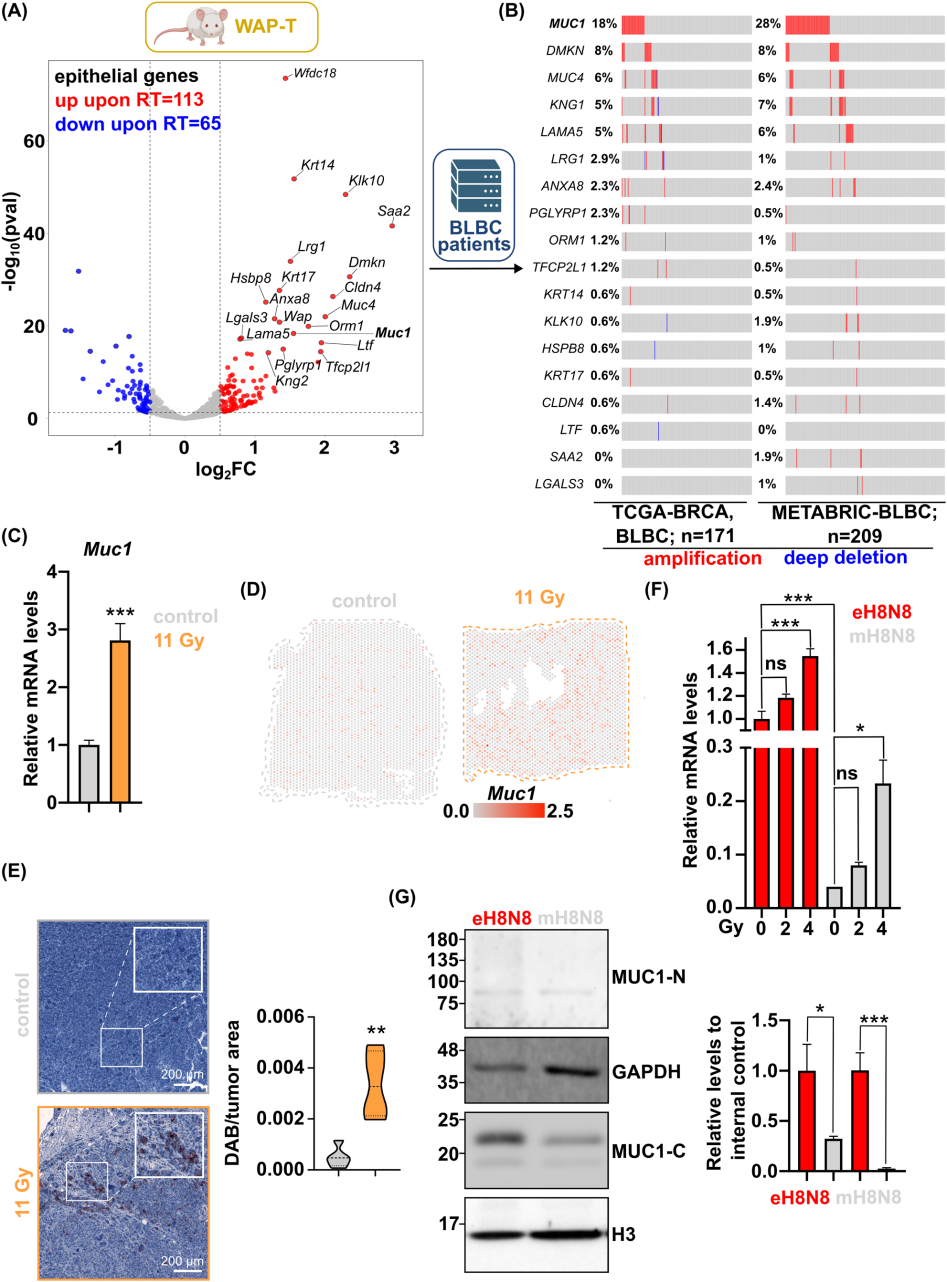
The expression of the epithelial marker MUC1 is highly induced upon RT in BLBC. (A, B) Volcano plot of differentially expressed epithelial genes in control and RT‐treated WAP‐T tumors (A). The top 20 highly expressed epithelial genes upon RT are circled and subsequently screened for copy number variations (CNV) in BLBC patients (B) using the cBioportal platform (*Source:* www.cbioportal.org). Source of epithelial gene list: www.panglaodb.se. (C) RT‐qPCR of *Muc1* in control and RT‐treated WAP‐T tumors. (D, E) Spatial transcriptomic expression of *Muc1* (D), and its immunohistochemical detection in control‐ and RT‐treated WAP‐T tumors (left panel) with its respective quantification (right panel, E). White scale bars: 200 μm. (F) RT‐qPCR of *Muc1* in control and RT‐treated eH8N8 and mH8N8 cells. (G) Protein analysis of MUC1‐C and MUC1‐N with their representative loading controls from one biological replicate (left panel) and the respective densitometric quantification (right panel) in eH8N8 and mH8N8 cells are shown. Statistical tests: (E) Mann–Whitney test; (F) One‐way ANOVA; (C, G) Student *t*‐test. ns: Not significant, *p < 0.05, **p ≤ 0.01, ***p ≤ 0.001. All experiments were performed in biological triplicates.

While the role of mesenchymal genes in promoting cancer cell aggressiveness is well‐established, the contribution of epithelial genes to RT resistance remains relatively unexplored. Emerging evidence, however, implicates hybrid states in the acquisition of therapy‐resistant phenotypes, including resistance to RT [25]. Given the stimulation of the epithelial transcriptional program upon RT, and the increased radiation resistance observed in eH8N8 cells, we sought to identify which factors within the epithelial gene signature might be responsible for this effect. First, we reanalyzed our bulk mRNA‐seq data, focusing on genes associated with epithelial phenotypes. *Muc1* emerged as a promising candidate, given its recent association with therapy resistance acquisition [26, 27, 28] and its prominent upregulation upon RT (Figure 4A). Additionally, analysis of publicly available copy number variation data from BLBC patients within the TCGA‐BRCA and METABRIC (Molecular Taxonomy of Breast Cancer International Consortium) datasets revealed a high frequency of *MUC1* amplification events compared to other candidate epithelial markers, supporting an important role of MUC1 for cancer cell fitness (Figure 4B). RT‐qPCR in the irradiated H8N8 tumors in vivo indeed validated the significant increase of *Muc1* upon RT (Figure 4C). Additionally, the results of the spatial transcriptomic dataset clearly identified an increase of *Muc1*‐positive areas in RT‐treated tumors (Figure 4D). Further immunohistochemical detection of MUC1 in the murine tumor cohorts corroborated the results obtained in the omic datasets (Figure 4E). Interestingly, exposure to RT strongly induced *Muc1* expression in H8N8 cells, whose levels remained constantly higher in eH8N8 than mH8N8 cells at basal conditions and under RT (Figure 4F).

MUC1 is a transmembrane glycoprotein that, after synthesis, is cleaved into two fragments, a large extracellular fragment (MUC1‐N) and a membrane‐anchored C‐terminal fragment (MUC1‐C) containing the transmembrane and cytoplasmic domains, forming a functional heterodimeric protein [29]. We first examined the expression of MUC1‐N and MUC1‐C in both H8N8 variants. We observed that MUC1‐C levels are higher than MUC1‐N in both eH8N8 and mH8N8 cells. This may be attributed to a hypoglycosylated, cancer‐specific form of MUC1 that affects its stability [30]. As expected, both fragments showed higher expression in eH8N8 cells, consistent with the epithelial specificity of MUC1 (Figure 4G). Several previous studies have reported that MUC1‐C can regulate oncogenic transcriptional programs associated with EMT [31]. We therefore asked whether RT‐mediated MUC1 induction underlies the induction of the epithelial gene signature. To address this, we silenced MUC1 using siRNA in eH8N8 cells, followed by RT‐qPCR analysis. Interestingly, MUC1 knockdown did not result in significant changes in epithelial or mesenchymal marker expression, suggesting that MUC1 acts as an epithelial‐specific effector rather than a master regulator of the epithelial transcriptome (Figure S2B).

Collectively, these findings indicate that MUC1 is a prominent epithelial marker whose expression is significantly increased in BLBC cells upon RT treatment, suggesting that it may contribute to their aggressiveness and enhanced survival.

### 3.5 MUC1 Supports the Aggressive Phenotype of BLBC Toward RT

**FIGURE 5:**
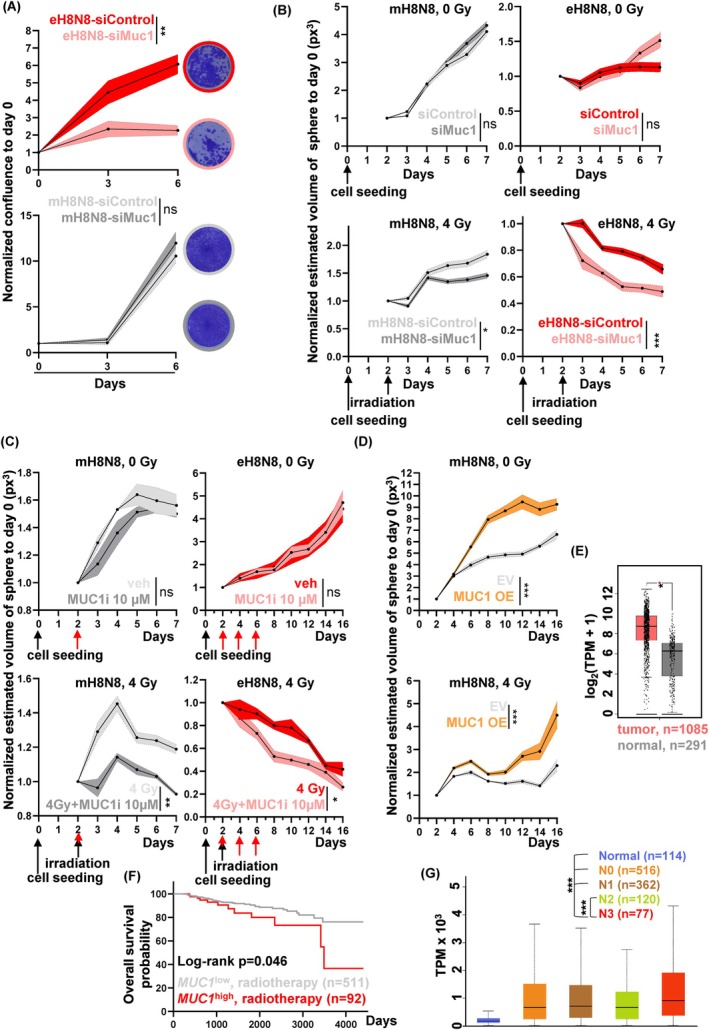
MUC1 supports the aggressive phenotype of BLBC toward RT. (A) Growth kinetics of siControl‐ and siMUC1‐treated eH8N8 and mH8N8 cells with the respective crystal violet staining at the last day of confluence measurement. (B, C) Growth kinetics of control and 4 Gy‐treated eH8N8 and mH8N8 spheres upon siControl and siMUC1 (B) or vehicle and MUC1i (MUC1i doses indicated in red arrows) at 10 μM (C). (D) Growth kinetics of empty vector (EV) and MUC1‐overexpressing (MUC1 OE) mH8N8 cells at basal growth conditions and upon 4 Gy. (E) *MUC1* expression in BC biopsies and healthy donors of mammary tissue (*Source:* http://gepia.cancer‐pku.cn/). (F) Overall survival probability analysis of *MUC1* ^low^‐ and *MUC1* ^high^‐expressing BC patients that received RT. (G) Box‐whiskers plot of *MUC1* expression (transcripts per million, TPM) in normal, N0, N1, N2 and N3 BC patients (*Source:* https://ualcan.path.uab.edu/). Statistical tests: (A–D) Student *t*‐test (based on the area under the curve, AUC); (E) Mann–Whitney test; (G) One‐way ANOVA, ns: Not significant *p < 0.05, **p ≤ 0.01, ***p ≤ 0.001. All experiments were performed in biological triplicates.

Relying on our previous results, we next wanted to investigate if MUC1 indeed supports the survival of BLBC cells upon RT. To further test the functional relevance of MUC1 in H8N8 cells, we performed several functional assays. We first investigated the consequence of MUC1 silencing on eH8N8 and mH8N8 cell growth in 2D conditions. Efficient MUC1 knockdown over time was confirmed by Western blotting and RT‐qPCR (Figure S2C,D). As expected, we observed that siRNA‐mediated MUC1 knockdown only impaired the growth kinetics of MUC1^high^ eH8N8 but did not influence MUC1^low^ mH8N8 cells (Figure 5A). Also under normal 3D growth conditions, both eH8N8 and mH8N8 spheres showed no reduced growth properties upon si*Muc1* treatment. However, and interestingly, upon RT treatment, MUC1 silencing enhanced growth inhibition both in eH8N8 and mH8N8 compared to control spheres with sole radiation (Figure 5B), aligning with the previously observed re‐expression of *Muc1* in mH8N8 in response to RT (Figure 4F). To enhance the translational relevance of our study, we used the MUC1‐specific inhibitor GO‐203 (MUC1i), which targets the CQC motif of MUC1‐C's cytoplasmic domain to block its pro‐tumorigenic activity [32]. Consistent with the radiosensitization observed upon MUC1 silencing in both eH8N8 and mH8N8 cells (Figure 5B), treatment with MUC1i (10 μM) sensitized both cell variants to radiation therapy (Figure 5C), supporting its therapeutic potential in BLBC. To further validate these findings, MUC1 was stably overexpressed in MUC1^low^ mH8N8 cells (MUC1 OE) (Figure S2E). Notably, MUC1 OE tumorspheres exhibited increased basal growth and enhanced resistance to radiation compared to empty vector controls (EV) (Figure 5D). In conclusion, given the higher basal expression of MUC1‐C than MUC1‐N in H8N8 cells (Figure 4F,G) and relying on the radiosensitizing function of the MUC1‐C‐specific GO‐203 inhibitor (Figure 5C), these findings validate the critical role of MUC1‐C in promoting radioresistance in BLBC cells under a hybrid state. To translate these observations to a clinical context, we analyzed patient‐derived data from the TCGA‐BRCA database, revealing that MUC1 is (i) significantly upregulated in BC tissues compared to normal mammary tissue, (ii) associated with poorer survival outcomes and reduced response to radiotherapy, and (iii) correlated with lymph node metastasis (Figure 5E–G).

Together, these results highlight MUC1 as a key epithelial‐specific effector that enables BLBC cells to tolerate radiotherapy. Furthermore, pharmacologic targeting of MUC1 represents a promising strategy to sensitize tumors to standard radiotherapy, potentially improving treatment response and patient survival.

## 4 Discussion

BLBC is an aggressive subtype of BC with poor prognosis and a high rate of recurrence. Standard treatment options for BLBC, including chemotherapy and RT, often yield suboptimal responses, leading to disease relapse and metastasis [33]. These clinical challenges underscore the need for novel therapeutic approaches optimized to the unique biology of BLBC. Intense research is ongoing to elucidate the mechanisms underlying RT resistance in BLBC. EMT, frequently associated with CSC traits, has become a significant contributor to this phenomenon. EMT provides tumor cells with enhanced plasticity and survival capabilities, thereby enabling them to evade chemotherapy‐ and RT‐induced cell death, as we and other groups have reported [11, 12, 34]. Our group has identified several therapeutic targets that can sensitize BLBC lesions to standard chemotherapy. These targets, including NFATC1 [11], HDAC8 [12], and AXL [13], play crucial roles in controlling phenotypic plasticity and maintaining CSC characteristics in BLBC cells. Specifically, chemotherapy‐induced loss of the repressive histone mark H3K27me3 leads to an NFATC1‐driven EMT, promoting drug tolerance and recurrence. Additionally, HDAC8‐mediated epigenetic silencing of transcription factors that oppose EMT supports drug tolerance and chemoresistance [12]. Furthermore, AXL is a key target to combat the CSC‐like subpopulation responsible for drug resistance and disease recurrence [13].

In various cancers, hybrid state cells have been associated with increased metastatic potential, therapy resistance, and poor patient outcomes [25, 35, 36, 37, 38, 39]. These cells can migrate collectively, forming tumor projections or circulating tumor cell clusters, which may enhance their ability to establish metastases [24]. In TNBC/BLBC, hybrid states are particularly prevalent and clinically significant. TNBC primary tumors often contain a higher percentage of hybrid state areas compared to other breast cancer subtypes [40]. The presence of hybrid state cells in TNBC is correlated with reduced patient relapse‐free survival and increased metastatic potential. In TNBC patient‐derived xenografts and organoids, hybrid state cells frequently lead to invasive strands, highlighting their role in tumor progression. Additionally, TNBC/BLBC tumors often exhibit molecular features associated with hybrid states, such as BRCA1 dysfunction, *TP53* mutations, and EGFR expression, which contribute to their aggressive behavior and poor prognosis [41]. Our present study surprisingly shows that resistance to RT in the WAP‐T BLBC model stimulates epithelial gene expression without notably affecting mesenchymal markers, resulting in a pronounced E/m‐state. This observation aligns with a recent single‐cell and spatial profiling study of TNBC biopsies from ICI‐ and RT‐treated patients, which reported that non‐responders had more epithelial cell clusters and fewer immune cells than responders, validated by higher pan‐cytokeratin and lower CD20, CD3e, and CD86 immunohistochemical markers [42]. Collectively, these findings underscore the importance of hybrid E/m states in TNBC/BLBC pathogenesis and suggest potential therapeutic targets for these challenging subtypes of breast cancer.

Epigenetic modifications and transcriptomic changes are central to the emergence of hybrid states. These regulatory mechanisms involve dynamic changes in chromatin structure, DNA methylation, and histone modifications that influence the expression of key regulators of mesenchymal (e.g., ZEB1, SNAIL, and TWIST) and epithelial (e.g., GRHL1/2 and OVOL1/2) phenotype [24]. The resulting hybrid state enhances tumor cell survival and metastatic potential, particularly in hostile microenvironments such as those encountered during RT [25, 35, 36, 37, 43, 44]. Moreover, the more mesenchymal e/M‐state phenotypes, which share similarities with the hybrid state, are implicated in RT resistance [45]. For instance, loss of CD24 expression leads to the repositioning of cells in a hybrid state, which displays increased stemness properties and intrinsic radio‐resistance through the downregulation of ROS levels and lower mitochondrial functions [46]. Additionally, EpCAM‐overexpressing cells in a E/m state show enhanced resistance to radiation‐induced DNA double‐strand breaks by activating key molecules in DNA repair pathways, such as Ku80 for non‐homologous end joining and Rad50 for homologous recombination, as well as activating ATM and its downstream target CHK2 [47].

MUC1, identified as a highly ranked epithelial marker in our models, exhibits a manifold increase upon RT exposure in both in vivo and in vitro settings. It has been reported that MUC1 expression is regulated by different transcription factors such as by Gata3 [29]. Also, Estrogen Receptor alpha (ER‐alpha) is involved in *MUC1* expression in BC [29]. This overexpression correlates with the promotion of the E/m‐state, facilitating RT resistance. Our findings are consistent with studies in other cancers, where MUC1 has been implicated in tumor progression and therapy resistance [29]. Notably, MUC1's role in hepatocellular, neuroendocrine prostate cancer, colon cancer, and head and neck squamous cell carcinoma further highlights its significance as a mediator of RT resistance [29].

Interestingly, we found that BLBC cells in an E/m‐state overexpressing MUC1 exhibit increased sensitivity upon MUC1 loss or inhibition. This finding highlights the therapeutic potential of targeting MUC1 to disrupt resistance mechanisms associated with radiation therapy. MUC1‐C inhibitors, such as GO‐203, have demonstrated efficacy in preclinical models including ours and especially when combined with RT, providing a foundation for combining these agents with standard therapeutic options to improve pathological response and patient survival outcomes [48, 49, 50]. Furthermore, a study in 2004 reported that indole‐3‐carbinol inhibits *MUC1* expression by activating AhR in BC [51]. The implications of our findings extend beyond BLBC as MUC1's involvement in RT resistance mechanisms across different cancer types has been extensively shown and which highlights its potential as a cancer‐wide therapeutic target [29]. Therefore, future studies should explore the translational application of MUC1 inhibitors in combination with RT, with a particular focus on identifying predictive biomarkers for patient selection.

While our WAP‐T model employs whole‐body irradiation unlike the localized radiotherapy in TCGA BLBC patients, the fundamental adaptive mechanisms activated in cancer cells in response to radiation stress such as epithelial‐mesenchymal plasticity and survival pathway induction, are presumably widely conserved, underscoring the relevance of our findings despite this methodological limitation. Additionally, concerning the clinical perspective of our findings, the development of combination therapies integrating MUC1 inhibitors and RT, with validation in clinical trials could be a highly promising therapeutic approach to test the findings of our current study in patients' response to RT. Finally, examination of MUC1‐mediated RT resistance across various cancer types could be of high interest to establish a cancer‐wide prognostic gene panel, given the high expression of *MUC1* in several cancer types compared to normal tissue counterparts (source: http://gepia.cancer‐pku.cn/).

In conclusion, our study identifies the E/m‐state and the subsequent upregulation of MUC1 as a critical mechanism underlying radiation resistance in BLBC. These findings pave the way for novel therapeutic strategies targeting MUC1 to overcome RT resistance and improve outcomes for patients with aggressive breast cancers.

## Author Contributions

**Garyfallia Pantelaiou‐Prokaki:** writing – original draft, conceptualization, investigation, methodology, validation. **Husam Bamahmoud:** investigation, validation, formal analysis. **Nadine S. Georges:** investigation, validation, formal analysis. **Shaishavi Jansari:** investigation, validation, visualization, writing – review and editing. **Annika Jung:** investigation, validation, formal analysis. **Daniela Grimm:** formal analysis, software, visualization. **Evangelos Prokakis:** visualization, writing – original draft, data curation, supervision. **Fabian Alexander Gayer:** resources. **Maryam Deldar:** investigation. **Christian Dullin:** investigation, validation, methodology. **Julia Gallwas:** writing – review and editing, conceptualization. **Florian Wegwitz:** conceptualization, investigation, software, formal analysis, visualization, writing – original draft, supervision, writing – review and editing, project administration, resources, validation. **Frauke Alves:** conceptualization, writing – original draft, writing – review and editing, funding acquisition.

## Funding

This research was funded by the Max‐Planck‐Institute for Multidisciplinary Sciences and in part by the Bundesministerium für Bildung und Forschung (BMBF)–AZ 13GW0218C.

## Ethics Statement

All animal in vivo procedures were performed in compliance with the guidelines of the European Directive (2010/63/EU) and in accordance with German regulations for animal experiments (Niedersächsisches Landesamt für Verbraucherschutz und Lebensmittelsicherheit, LAVES, ethics approval no. AZ33.9‐42502‐04‐18/3022).

## Conflicts of Interest

The authors declare no conflicts of interest.

## Supporting information


**Table S1:** siRNAs.
**Table S2:** Primers for RTqPCR.
**Table S3:** PCR program for RTqPCR.
**Table S4:** Components of the master mix for qPCR.
**Table S5:** Protease inhibitors used in RIPA buffer.
**Table S6:** Antibodies (WB: western blot, IF: immunofluorescence, IHC: immunohistochemistry).
**Table S7:** RNA sequencing (RNA‐seq) coverage and quality statistics.
**Table S8:** spatial RNA sequencing (spRNA‐seq) coverage and quality statistics.
**Figure S1:** (A) Gene expression heatmap of epithelial and mesenchymal genes in control and RT‐treated WAP‐T tumors. (B) Spatial transcriptomic expression of Icam1, Cldn4, Snai2, and Tgfb2 in control and RT‐treated WAP‐T tumors. (C) Immunofluorescence staining of KRT18 and KRT14 in control and 11 Gy‐treated WAP‐T tumors (left panel) and their respective quantification (right panel). DAPI is used as counterstain. White scale bars in C: 200 μm. Statistics: C Student *t*‐test, ***p ≤ 0.001. All experiments were performed in biological triplicates.
**Figure S2:** (A) RT‐qPCR of *Ovol1, Grhl2, Sox9, Cebpd*, in control, 2 Gy, and 4 Gy‐treated eH8N8 and mH8N8 cells. (B) RT‐qPCR of several epithelial and mesenchymal markers in siControl and *siMuc1*‐treated eH8N8 cells. siControl‐derived expression of the genes is depicted as a relative mean demarcated by the dashed line. (C) RT‐qPCR of *Muc1* in siControl and siMUC1‐treated eH8N8 cells at 72, 96, and 120 h post‐transfection. (D) Western blotting of MUC1‐C and MUC1‐N and their respective internal loading controls from one representative biological replicate in siControl‐ and siMUC1‐treated eH8N8 cells at 72, 96, and 120 h post‐transfection (upper panel), as well as the corresponding densitometry analyses (lower panel). (E) RT‐qPCR of *Muc1* in EV and MUC1 OE mH8N8 cells. Statistics: A–E Student *t*‐test; ns: not significant, *p < 0.05, **p ≤ 0.01, ***p ≤ 0.001. All experiments were performed in biological triplicates.

## Data Availability

High‐throughput sequencing data have been deposited in ArrayExpress (source: https://www.ebi.ac.uk/biostudies/arrayexpress/studies) under the following accession numbers and links: (1) Title: “mRNA‐seq of H8N8 derived mammary tumors, untreated (Ctr.) or upon 11 Gy total dose (Rad.)”, Accession number: E‐MTAB‐14986. (2) Title: Basal‐like breast cancer stimulates MUC1 to support an epithelial/mesenchymal hybrid state and survive radiotherapy, Accession number: E‐MTAB‐15010. Further information is available from the corresponding author upon request.
