# Supplementary material for: MUC1 as a Survival Effector of Radiotherapy‐Induced Epithelial Hybrid States in Basal‐Like Breast Cancer
Source: Int J Cancer. 2026 Apr 18;159(5):1296–310. doi: 10.1002/ijc.70487 (PMC13340933; doi:10.1002/ijc.70487)
Supplement: Supplementary file 1 — Table S1: siRNAs. Table S2: Primers for RTqPCR. Table S3: PCR program for RTqPCR. Table S4: Components of the master mix for qPCR. Table S5: Protease inhibitors used in RIPA buffer. Table S6: Antibodies (WB: western blot, IF: immunofluorescence, IHC: immunohistochemistry). Table S7: RNA sequencing (RNA‐seq) coverage and quality statistics. Table S8: spatial RNA sequencing (spRNA‐seq) coverage and quality statistics. Figure S1: (A) Gene expression heatmap of epithelial and mesenchymal genes in control and RT‐treated WAP‐T tumors. (B) Spatial transcriptomic expression of Icam1, Cldn4, Snai2, and Tgfb2 in control and RT‐treated WAP‐T tumors. (C) Immunofluorescence staining of KRT18 and KRT14 in control and 11 Gy‐treated WAP‐T tumors (left panel) and their respective quantification (right panel). DAPI is used as counterstain. White scale bars in C: 200 μm. Statistics: C Student t‐test, ***p ≤ 0.001. All experiments were performed in biological triplicates. Figure S2: (A) RT‐qPCR of Ovol1, Grhl2, Sox9, Cebpd, in control, 2 Gy, and 4 Gy‐treated eH8N8 and mH8N8 cells. (B) RT‐qPCR of several epithelial and mesenchymal markers in siControl and siMuc1‐treated eH8N8 cells. siControl‐derived expression of the genes is depicted as a relative mean demarcated by the dashed line. (C) RT‐qPCR of Muc1 in siControl and siMUC1‐treated eH8N8 cells at 72, 96, and 120 h post‐transfection. (D) Western blotting of MUC1‐C and MUC1‐N and their respective internal loading controls from one representative biological replicate in siControl‐ and siMUC1‐treated eH8N8 cells at 72, 96, and 120 h post‐transfection (upper panel), as well as the corresponding densitometry analyses (lower panel). (E) RT‐qPCR of Muc1 in EV and MUC1 OE mH8N8 cells. Statistics: A–E Student t‐test; ns: not significant, *p < 0.05, **p ≤ 0.01, ***p ≤ 0.001. All experiments were performed in biological triplicates. [file IJC-159-1296-s001.pdf]

Supplementary Information

MUC1 as a survival effector of radiotherapy-induced epithelial hybrid states in basal-like breast cancer

Garyfallia Pantelaiou-Prokaki, Husam Bamahmoud, Nadine S. Georges, Shaishavi Jansari, Annika Jung, Daniela Grimm, Evangelos Prokakis, Fabian Alexander Gayer, Maryam Deldar, Christian Dullin, Julia Gallwas, Florian Wegwitz, Frauke Alves

Table of contents

Cell culture 2
Crystal violet staining 2
Immunohistochemistry and quantification 2
Establishment of 3-dimensional spheres in U-Bottom 96-well plate for MUC1i- and siMUC1-based functional experiments 2
siRNA transfection 3
RNA isolation 4
RNA quality check 4
cDNA synthesis 5
RTqPCR 5
Protein extraction and western blotting 7
Cryopreservation, cryosectioning of tumor tissues and immunofluorescence staining and quantification 9
Cryopreservation and cryosectioning of H8N8 spheres 9
Immunofluorescence from cryopreserved H8N8 spheres and quantifications 10
Supplementary Figures 11
    Figure S1 11
    Figure S2 12

List of tables

Table S1: siRNAs 3
Table S2: Primers for RTqPCR 6
Table S3: PCR program for RTqPCR 7
Table S4: Components of the master mix for qPCR 7
Table S5: Protease inhibitors used in RIPA buffer 8
Table S6: Antibodies (WB: western blot, IF: immunofluorescence, IHC: immunohistochemistry) 8
Table S7: RNA sequencing (RNA-seq) coverage and quality statistics 10
Table S8: spatial RNA sequencing (spRNA-seq) coverage and quality statistics 10

Cell culture

The murine eH8N8 and mH8N8 cell line was cultured in three biological replicates using Dulbecco's Modified Eagle Medium (DMEM) with glucose (4.5 g/L). The medium was supplemented with 10% FBS and 1% penicillin-streptomycin (10,000 units/ml penicillin and 10,000 µg/ml streptomycin). Cultures were maintained at 37°C in a 5% CO_2 atmosphere. For passaging, H8N8 cells were washed once with 1xPBS and trypsinized for 5 min at 37°C using 0.05% Trypsin-EDTA for mesenchymal cells and 0.25% Trypsin-EDTA for epithelial cells. Cell counting was performed three times using a CellDrop™ followed by manual counting with a Neubauer counting chamber for reference.

Establishment of the MUC1-OE clone: mH8N8 cells were transfected with pSB[Exp]-CMV>mMuc1[NM_013605.2](ns):P2A:EGFP:T2A:Puro (VectorBuilder, VB240813-1192ygd) and the sleeping beauty transposition system SB-Transposase plasmid (VectorBuilder, VB180507-1151nqp) using the lipofectamine 2000 reagent (Thermofisher), according to the manufacturer's instructions. pSB[Exp]-CMV>EGFP:T2A:Puro/SB-transposase (empty vector; EV cells) and pSB[Exp]-CMV>mMuc1[NM_013605.2](ns):P2A:EGFP:T2A:Puro/SB-transposase (MUC1 OE) co-transfected mH8N8 cells were GFP-sorted and seeded in single cells in a 96-well plate for clonal expansion. Finally, MUC1 OE cells were validated via RTqPCR for *Muc1* overexpression versus EV cells.

Crystal violet staining

Cells in culture plates were rinsed once with PBS, then fixed with 100% methanol for 10 min at room temperature. A 1% crystal violet solution dissolved in 20% methanol was applied to stain the cells for 20 min at room temperature. Plates were then washed with water to remove excess stain, dried overnight, and scanned using an EPSON Perfection V850 Pro. Scanned images were analyzed with ImageJ.

Immunohistochemistry and quantification

The 5 µM paraffin-embedded sections were initially incubated at 60°C for 10 min, followed by a 20-min incubation in xylene. They were then immersed in a 50:50 xylene solution for 10 min before undergoing a graded ethanol series (100%, 90%, and 70%) for 5 min at each concentration. After rinsing with tap water for 5 min, the slides were microwaved for 10 min for antigen retrieval and allowed to cool for 5-10 min. Subsequently, the slides were washed three times in phosphate-buffered saline (PBS) for 3 min each and blocked at room temperature for one hour in 5% bovine serum albumin (BSA) in PBS. The slides were then incubated overnight at 4°C with the primary antibody (Table S6) in 5% BSA and 1:100 diluted donkey serum. The following day, the slides were washed three times with PBS containing Tween-20 (PBS-T) for 5 min each and incubated for one hour with a biotinylated secondary antibody (1:200 in 5% BSA and 1:100 diluted donkey serum, Table S6). After washing with PBS-T and PBS, the slides were incubated for 90 min with avidin (1:800 in PBS) and subsequently washed with PBS. Signal development was monitored under a microscope using DAB until the desired signal was detected. The slides were counterstained with hematoxylin for 5-7 min, rinsed with an ethanol series, and mounted with a medium before drying overnight. DAB signal area was normalized to tumor area (areas were calculated using Fiji v1.54m) and results were graphed using Prism v8.0.1.

Establishment of 3-dimensional spheres in U-Bottom 96-well plate for MUC1i- and siMUC1-based functional experiments

To study tumors *in vitro*, we utilized the well-known method of tumorsphere formation. The 3D tumorsphere model is an effective and straightforward approach that closely mimics the three-dimensional growth conditions of tumors which takes place *in vivo*, enabling a more accurate and realistic study of tumor characteristics such as the cancer stem cell (CSC) population in vitro than a conventional monolayer 2D culture. Ultra-low attachment U-bottom 96-well plates were used for this purpose (Corning, Clear round bottom ultra-

low attachment cat. 7007). Using U-bottom ultra-low attachment plates allows cells to come into close proximity, facilitating the formation of a single sphere within an efficient time frame. For murine H8N8 cells, it took approximately 48 hours after seeding to form a single sphere, which was then treated with the GO-203 (Selleckhem; MUC1i) at 10 µM and 2, 4, or 6 Gy radiation. For MUC1i-treated eH8N8 cells, cells were initially treated in 2D with MUC1i at 10 µM for 24 hours, subsequently seeded in 96-well U-bottom plates, treated with 4 Gy at 48 hours after seeding and replenished with MUC1i every 48 hours for up to three cycles. For MUC1i-treated mH8N8 cells, cells were directly seeded on the same type of plate, and 4Gy or/and MUC1i (10µM) was applied 48 hours after seeding without further inhibitor replenishment and their growth kinetic monitoring lasted in total for 9-14 days. For siRNA-based functional assays in 3D tumorsphere settings, eH8N8 and mH8N8 cells were reverse transfected with siControl or siMUC1 in 2D culture according to the manufacturer’s instructions (see next ‘‘siRNA transfection’’), and at 48 hours post-transfection cells were seeded on the 96-well U-bottom plates. Finally, like in MUC1i-based experiments, RT was applied 48 hours after seeding and monitored for their growth kinetic for up to 5 days.

Technically, eH8N8 and mH8N8 cells were trypsinized and resuspended in fresh DMEM growth medium. Cell counting was performed as previously described using a CellDrop™ and a Neubauer counting chamber. Various cell numbers, ranging from 2000 to 24000 cells, were seeded into an ultra-low attachment U-bottom 96-well plate. After seeding, the plate was centrifuged at 125-300 g at room temperature (20-25°C). On the next day the plates were then scanned daily with Celigo and/or Incucyte to monitor the growth kinetics of the spheres. The spheres were scanned and monitored for the timeline of 5-14 days and the analysis of the tumor spheroids was done using ImageJ software by normalizing each spheroid to day 0.

siRNA transfection

The transfection mix was prepared as follows: For each well, 500 µl of OptiMEM, 5 µl of RNAiMax, and 1.5 µl of siRNA (either MUC-1-targeted SmartPool or non-targeting siRNA, Table S1) were combined in a 2 ml Eppendorf tube. The mixture was vortexed for 15-20 sec to ensure thorough mixing, then incubated at room temperature for 20 min. H8N8 epithelial and mesenchymal cells were trypsinized as previously described, and 200,000 cells were resuspended in 1.5 ml of DMEM growth medium. After incubation, 500 µl of the transfection mix was added to each well of a 6-well plate, followed by the addition of the cell suspension. The plate was gently shaken to ensure even distribution of the cells within each well. The cells were incubated with the transfection mix overnight. The next day, the medium was replaced with fresh DMEM growth medium, and the plate was incubated for an additional 72, 96 or 120 hours before proceeding with RNA or protein extraction.

For sphere formation, transfection was performed on the first day, and the medium was replaced 24 hours later. The fresh medium was then incubated for several hours before trypsinizing the cells again and seeding them as spheres in an ultra-low adherent 96-well plate as mentioned above.

Table S1: siRNAs

primer	Sequence	Cat-no/Company
Non-targeting #5 (NT5)	UGGUUUACAUGUCGACUAA	D-001210-05-50 Dharmacon
si*Muc1*	CAACAGCUCUCCAGUAGUC	D-042344-01 Dharmacon
	CUACCAGUCUAGUCUAUAA	D-042344-02 Dharmacon
	CCUGAAGACUCUACCAGUA	D-042344-03 Dharmacon
	AGUCACAGCUUAUACAGCA	D-042344-04

		Dharmacon

RNA isolation

From cell culture:

Total RNA was extracted from murine H8N8 cells using the NucleoSpin® RNA isolation kit according to the manufacturer's instructions. Plates were immediately placed on ice after removal from -80°C storage. Subsequently, 600 µl of lysis buffer RAV1 was added to each well, and the contents were pipetted up and down several times to ensure complete cell lysis, followed by a 3-min incubation at room temperature. Next, 600 µl of 100% ethanol was added to the cell lysate and mixed thoroughly. The 600 µl cell-lysate mixture was then loaded stepwise onto the isolation column and centrifuged at 8,000 x g, allowing the RNA to bind to the silica membrane. Following this, the column was washed with 500 µl of washing buffer RAW, followed by two additional washes with washing buffer RAW3, each centrifuged at 8,000 x g. To remove any residual washing buffer, a final centrifugation at 11,000 x g was performed. The pure RNA was then eluted with 50 µl of RNAase-free water and centrifuged for 2 min at 11,000 x g, and the RNA was collected into new, RNAase free clean, autoclaved Eppendorf tubes. The concentration of the RNA was then measured using a Nanodrop.

From tissue:

RNA from WAP-T NP8 mammary carcinomas were extracted using a Trizol/Chloroform protocol according to manufacturer´s instructions. For this purpose, shock frozen mammary tumors from female mice were used, which were stored at -80°C. To extract the RNA, approximately 40 mg of frozen tissue was cut for each condition on a dry ice-cooled block with a scalpel. Experiments were conducted with two untreated tumor tissues and four irradiated tumors. All utensils used for the preparation were disinfected with 70 % DEPC-EtOH. Tissues were collected in separate tissue-homogenization tubes filled with 1.4 mm ceramic beads. All the following steps were carried out on ice. 150 µl RIPA lysis buffer was added to the tubes in an ice-cold tissue-lyser and the tubes were shaken for 10 sec at 50 Hz. 1 ml (EXTRAzol) reagent was added, and the tissues were dissociated in the tissue lyser for three cycles 20 sec at 50 Hz and 30 sec break on ice. After centrifugation (12,000 x g, 1 min, 4 °C), the supernatant was transferred to new tubes and the samples were incubated for 5 min at room temperature to dissociate the nucleotide complexes. For phase separation, 200 µl chloroform was added and the tubes were vortexed for 15 sec. The lysate was incubated at room temperature for 10 min, followed by centrifugation (12,000x g, 10 min, 4°C). The resulting upper aqueous phase containing the RNAs was transferred to a new 1.5 ml tube and the second round of chloroform extraction was performed, adding again 200 µl chloroform, incubating 5 min at room temperature, centrifuging (12,000 x g, 5 min, 4 °C) and transferring the upper phase to a new 1.5 ml tube. The fresh tubes with 500 µl isopropanol and the upper phases were strongly mixed and incubated at -80 °C for at least 1 hour to precipitate the RNA. After centrifugation (12,000 x g, 10 min, 4 °C), the supernatant was discarded and the RNA pellet was washed with 80 % ice-cold EtOH (centrifugation 12,000 x g, 10 min, 4°C) and was centrifuged again (8,000 x g, 1 min, 4 °C) before air drying to remove excess EtOH. The RNA pellet was resuspended in 50 µl DEPC-H2O and RNA concentration was measured with DeNovix Spectrophotometer. To improve the quality of the mentioned RNA, a second cycle of RNA isolation was performed, following all the above-mentioned steps for EXTRAzol/Chloroform extraction.

RNA quality check

Electrophoresis is a widely used technique in molecular biology for identifying, quantifying, and purifying nucleic acid fragments, as well as assessing their quality. RNA molecules, having a negative charge, migrate

toward the positive anode when subjected to an electric current during gel electrophoresis. The migration of RNA in the gel is generally determined by its length, with the longer RNA molecules moving more slowly than shorter fragments. When an intact total RNA is run on an agarose gel, it will display sharp 28S and 18S rRNA bands in eukaryotic samples in our case the murine total-RNA isolated from H8N8 cells. The 28S rRNA band should be approximately twice as intense as the 18S rRNA band. This 2:1 ratio (28S:18S) is a strong indicator that the RNA isolated is intact and not degraded. For the electrophoresis the chambers were washed with soap and water followed by 70% ethanol and lastly rinsed with DEPC-H_2O and left to covered to dry. 1% agarose gel was prepared by dissolving 0.5g of agarose in 50 ml of 0.5X TBE buffer and heating it for approximately 2 min at 500 W. After cooling, 2.5 µl of HD Green was added to the gel under a fume hood. The cooked agarose solution was then poured into the chamber and the comb was placed after pouring the gel and the gel was allowed to solidify for 20 min. Meanwhile, 100 ml of 0.5X TBE buffer was prepared to fill the chamber and covering the agarose gel. RNA samples were prepared by mixing µl of DEPC-treated water with, 1 µl of loading dye, and 1 µl of RNA (approximately 400 ng/µl). These samples were denatured by shock-heating at 70°C for 2 min and then immediately placed on ice. A DNA ladder (100-3000 bp rainbow ladder) was used as a size marker. For loading, 5 µl of the DNA ladder, 10 µl of RNA samples, and 1 µl of loading dye were pipetted into consecutive wells. Electrophoresis was carried out at 120 V for 30 min. The gel was visualized under UV light at 312 nm using a BioDoc Analyze Transilluminator and a CCD video camera.
Alternatively, RNA quality was examined with the 2100 Bioanalyzer using the Agilent RNA 6000 Nano Kit (both Agilent Technologies, Santa Clara, CA, US) for RNA-sequencing and with RNA gel electrophoresis for ordinary RT-qPCR. cDNA synthesis was performed using the reverse transcriptase kit from New Englands Biolabs®.

cDNA synthesis

cDNA synthesis was performed using the reverse transcriptase kit from New Englands Biolabs®. The cDNA synthesis from RNA was performed by ensuring that 500 ng or 1000 ng of RNA was mixed with 2 µl of random primers, 1 µl of dNTPs, and DEPC-treated water to a total volume of 10 µl in an RNase-free Eppendorf tube. The tube was then incubated at 65°C for 5 min and immediately placed on ice. Subsequently, 1 µl of reverse transcriptase (RT) at 200 U/µl, 0.2 µl of RNase inhibitor at 40 U/µl, 2 µl of 10X M-MulV buffer, and nuclease-free water were added to reach a final volume of 20 µl. The mixture was incubated at 25°C for 5 min, followed by 42°C for 1 hour. The reaction was terminated by heating at 65°C for 20 min. The synthesized cDNA was then diluted with H_2O and stored at -20°C.

RTqPCR

Real-Time q-PCR, also known as quantitative PCR (qPCR), is a method used for the detection and quantification of DNA. Real-time PCR methods utilize the real-time measurement of fluorescence from a dye that specifically binds to double-stranded DNA (dsDNA), in our case SYBR® Green. This dye shows a weak background fluorescence, which increases and accumulates upon binding to dsDNA. The amplification of the target gene sequence results in an increase in fluorescence that is directly proportional to the amount of dsDNA present in each PCR cycle. This type of real-time PCR requires only two for the gene of interest sequence-specific primers (Table S2), the forward and the reverse primer. The samples were normalized to the *Rplp0* or *18s rRNA* housekeeping gene. A more detailed protocol is provided in Supplementary Information. For our purpose we made use of this easy and efficient method to analyze our genes of interest.
Briefly, each qPCR reaction (Table S3-4) was prepared as follows: 1 µl cDNA, 14 µl of homemade PCR master mix (exact components of the master mix in Table S4), 9 µl of H2O, and 1 µl of primer (1:100) were pipetted into a 96-well plate.
A standard curve was generated for each primer pair to quantify relative gene expression. To create the starting concentration (1:1), equal volumes from all samples were pooled. Subsequent concentrations (1, 1/4, 1/16, 1/64, 1/256) were prepared by serially diluting the 1/1 concentration with molecular-grade H_2O. The standard curve was run in technical duplicates, while each sample was analyzed in technical triplicates. The

plate was covered with an adhesive plastic foil and centrifuged briefly at 300 xg. Finally, the plate was measured using the Bio-Rad CFX Connect Real-Time System following the specified protocol from Bio-Rad©. Samples were run with the following amplification program: 95°C for 2 min, 40 cycles (95°C for 15 sec, 60°C for 30 sec, Table S3) in a BioRad CFX96 system (USA). A melting curve analysis was subsequently generated (67°C-95°C, 0.5°C/sec).

Table S2: Primers for RTqPCR

primer	Sequence
Rplp0	Forward: GCTGAACATCTCCCCCTTCT<br>Reverse: CCCATTGATGATGGAGTGTG
18s rRNA	Forward: CGGACAGGATTGACAGATTG<br>Reverse: CAAATCGCTCCACCAACTAA
Ovol1	Forward: ACGTGCAAGAGGAACTGGAG<br>Reverse: AAGGCTCATGTCCAAAGCCA
Grhl2	Forward: AAAAGGGGAGCGAGTTCATT<br>Reverse: GTAGGCTCTTCGGGTGTTGA
Sox9	Forward: CGGAGCTCAGCAAGACTCTG<br>Reverse: TCCGTTCTTCACCGACTTCC
Cebpd	Forward: GCACAGTCCGAGAAAAGGGC<br>Reverse: ACCAGCTTCTGCTGCATCTC
Cdh2	Forward: AGAGCACATGCAGTGGACATC<br>Reverse: GGCAGTGACCGTCATCACATA
Snai2	Forward: AACATTTCAACGCCTCCAAG<br>Reverse: CGAGGTGAGGATCTCTGGTT
Zeb1	Forward: CCAAGAACTGCTGGCAAGACAA<br>Reverse: ATGACTGCTGGCTTCTGGTGTG
Cdh1	Forward: AGCCATTGCCAAGTACATCCTC<br>Reverse: GGCCTGTTGTCATTCTGATCTG
Krt17	Forward: CATCCTGCTCCAGATTGACA<br>Reverse: GCCAGCTCCTCCTTGAGATT
Krt18	Forward: TCTGGGATGCAGAACATGAG<br>Reverse: TCTTCACAACCACAGCCTTG
Krt8	Forward: TCTGGGATGCAGAACATGAG<br>Reverse: TCTTCACAACCACAGCCTTG
Muc1	Forward: TGCTGGTGCTGGTCTGTATT<br>Reverse: TCCTGGGTTGGAAAGATGTC
Tjp3	Forward: CCATGACCGAGCTAGTGGAT<br>Reverse: GCTGTTTTGGTGCAGGTCTT
Vim	Forward: CGGCTGCGAGAGAAATTGC<br>Reverse: CCACTTTCCGTTCAAGGTCAAG

Table S3: PCR program for RTqPCR

Step	Temperature (°C)	time	Cycles
1	95	2 min	1
2	95	10 sec	40
	60	30 sec	1
3	60 to 95 (0.5 increments)	1 sec	1
4	95	5 sec	1
5	4	Hold	1

Table S4: Components of the master mix for qPCR

Components for 10X buffer	Stock Concentration	volume
$(NH_4)SO_4$	1 M	8.0 ml
H_2O (for molecular biology)		100 µl
Tris-HCL (pH8.8)	1.5 M	5 ml
Tween-20	10%	7.5 ml
**Components for 2X qPCR-Mix**	**Stock Concentration**	**Volume**
10X buffer	10X	2.5 ml
dNTPs	20 mM	250 µl
$MgCl_2$	25 M	3 ml
Sybr Green	1:100	46.9 µl
Taq-polymerase	5 U/ µl	100 µl
Trehalose	1 M	7.5 ml
Triton X-100	10 %	625 µl

Protein extraction and western blotting

For protein extraction, 100,000 epithelial and 80,000 mesenchymal H8N8 cells were seeded into each well of a 6-well adherent plate. The cells were allowed to adhere for 48 hours. After this period, the cells were exposed to different doses of radiation (2, 4, and 6 Gy). Following radiation exposure, the cells were treated with 10 µM of the MUC-1 inhibitor (Selleckchem GO-203, Catalog No. S8674).

Seventy-two hours after treatment, the cells were washed twice with PBS to remove any media and cell debris. The cells were then lysed using 200 µl of RIPA lysis buffer supplemented with protease inhibitors (Table S5).The lysis buffer was added to each well, ensuring it covered the entire surface area, and the plates were incubated on ice at 4°C for 10 min. The cells were scraped with a cell scraper and transferred to clean 1.5 ml Eppendorf tubes, which were immediately placed on ice at 4°C.

The cell lysates were then subjected to sonication for 10 min (Bioruptor Plus) with 30-sec on/off cycles. After sonication, protein concentration was determined using the BSA kit from Thermo Scientific Pierce™ (Prod #23225) according to the manufacturer's instructions. A (1:50) solution was prepared by mixing 1 part B reagent with 50 parts A reagent. For each reaction, 100 µl of this working solution was pipetted into clean Eppendorf cups, followed by the addition of 2.5 µl of cell lysate. The mixture was incubated at 37°C and 1000 rpm for 30 min. After incubation, the samples were transferred to cuvettes and subjected to colorimetric measurement using the Colorimetric application on the NanoDrop. The cell lysates were diluted accordingly in Laemmli buffer and heated at 95°C for 5 min. The samples were then ready to be loaded on the gel.

Proteins were extracted from 6-wells plates with 500 µL ice-cold RIPA buffer containing protease inhibitors (Table S5) and following the standard procedure established in our lab. Equal amounts of samples were separated using 8 to 15% SDS polyacrylamide gel electrophoresis and transferred onto nitrocellulose membranes (Immobilon, Millipore, USA). After overnight incubation with the first antibody at 4°C and the HRP-coupled secondary antibody for one hour at room temperature (Table S6), the membranes were developed in a ChemoStar imaging system (INTAS science imaging, DE).

Table S5: Protease inhibitors used in RIPA buffer

Component (Protease inhibitor)	Working Concentration
A/L (Aprotinin/Leupeptin)	5 µg/ml
Activated orthovanadate	1 mM
BGP (Glycerol 2-phosphate)	10 mM
IAA (Iodoacetamide)	10 µM
NaF (sodium fluoride)	1 mM
NEM (N-Ethylmaleimide)	1 mM
Pefabloc	1 mM

Table S6: Antibodies (WB: western blot, IF: immunofluorescence, IHC: immunohistochemistry)

**Antibody**	**Dilution**	**Host**	**Cat.no**	**Company**
**GAPDH**	1:1000 (WB)	Rabbit	D16H11	Cell Signaling
**GAPDH hFAB**	1:5000 (WB)	-	12004168	BioRad
**H3**	1:1000 (WB)	Rabbit	601902	BioLegend
**a-tubulin hFAB**	1:5000 (WB)	-	12004166	BioRad
**MUC1**	1:250 (IHC, antigen retrieval: Tris-EDTA buffer, pH 9.0)	Rabbit	Ab109185	Abcam
**MUC1**	1:1000 (WB)	Rabbit	A0333	ABclonal
**γH2Ax**	1:250 (IHC, antigen retrieval: Sodium Citrate Buffer (10mM Sodium Citrate, 0.05% Tween 20, pH 6.0))	Rabbit	#9718	Cell Signaling
**Krt14 (AF64)**	1:200 (IF)	Rabbit	PRB155P100	Covance
**Krt18**	1:100 (IF)	Mouse	61028	Progen Biotechnik
**E-cadherin**	1:100 (IF); 1:1000 (WB)	Rabbit	3195S	Cell Signaling
**Vimentin**	1:100 (IF)	Chicken	NB300-223	Novus Biologicals
**Vimentin**	1:1000 (WB)	Rabbit	D21H3	Cell Signaling
**Biotin-anti-rabbit IgG**	1:200 (IHC)	Donkey	711-065-152	Dianova

**Anti-rabbit IgG-HRP**	1:10000 (WB)	Donkey	715-035-150	Jackson Labs
**Anti-rabbit (AlexaFluor 488)**	1:400 (IF)	Goat	A-11034	Mol. Probes
**Anti-chicken (AlexaFluor 555)**	1:400 (IF)	Goat	A-21437	Mol. Probes
**Anti-mouse (AlexaFluor 488)**	1:400 (IF)	Goat	A-11029	Mol. Probes
**Anti-rabbit (AlexaFluor 555)**	1:400 (IF)	Goat	A-21429	Mol. Probes

Cryopreservation, cryosectioning of tumor tissues and immunofluorescence staining and quantification

10 µm thick cryosections of irradiated and non-irradiated H8N8 mammary tumors were cut, transferred on superfrost glass slides and stored at -20 °C. For Immunofluorescence, sections were acclimated at room temperature for 30 min before fixation with 4 % PFA diluted in 1X PBS and permeabilized with 1 % Triton-X-100 in PBS for 5 min at room temperature. Specimens were blocked with blocking buffer (0.5 % BSA in PBS and 1 % normal donkey serum) for 1 hour at room temerature before incubation with the primary antibody overnight at 4 °C. Slides were washed twice with PBS-T and once with PBS each 5 min, followed by at least 2 hour incubation time with the fluorochrome-conjugated secondary antibody at room temerature in the dark. After further washing steps with PBS-T and PBS, DAPI was applied for 30 min at room temperature. Finally, the slides were washed with PBS and mounted with a Mowiol-mounting medium. Slides were stored the next day at -20 °C in the dark. For fluorescence signal quantification, the ratio of E-cadherin and Krt14-positive cells was calculated and divided by the number of nuclei (DAPI, blue) per field in 3 fields per condition. Quantifications were performed using Fiji v1.54m and graphed using Prism v8.0.1.

Cryopreservation and cryosectioning of H8N8 spheres

H8N8 Epithelial and mesenchymal spheres (3000 cells per sphere) were collected in 1.5 ml Eppendorf tubes and centrifuged at 300 x g, carefully discarding the supernatant to retain the spheres. A cryopreservation medium was prepared by mixing equal parts of culture medium and cryopreservation medium. Cryo freezing matrices were created by pouring the cryomatrix medium into molds and allowing them to solidify on dry ice. Once solidified, small indentations were made in the blocks to hold 20-50 µl of the sphere-containing solution. The cryopreservation medium containing the spheres was then pipetted into these cavities, ensuring no air bubbles formed. The blocks were placed directly on dry ice to solidify. Subsequently, a layer of cryomatrix was pipetted over the spheres to coat and protect them from freezing shock. The blocks were then placed back on dry ice to solidify completely. The cryo-blocks containing the spheres were then stored in -20°C.

To prepare the Cryotome, it was cooled overnight to -22 °C for the cryostat and -24 °C for the specimen head, with colder settings applied for more fatty tissues. The sample holder was positioned as far from the blade holder as possible, and paper towels were placed at the bottom of the machine for easier cleanup of cryomatrix residue. A new blade was inserted with the glass part left open. The cryomatrix was then used to fixate the sample pill to the stamp, filling any gaps slightly. The stamp, with the sample attached, was placed in the holder and moved forward toward the blade, ensuring the sample was aligned as parallel to the blade as possible. The sample was trimmed in increments of 30 to 80 µm until an even surface was achieved and the sample began to appear in the pill, marked by a yellow medium-matrix mix with the spheres. Following this, the blade was covered with glass, and the samples were sectioned at a thickness of 8 to 15 µm. The sections were held down with a brush to prevent rolling, and if rolling occurred, the sample was gently unrolled using two brushes after opening the glass part. A glass slide was used ultra frost slides for immunofluorescence and

regular slides for training or orientation. The sample was allowed to melt onto the slide, making the spheres visible on the surface of the slide.

Immunofluorescence from cryopreserved H8N8 spheres and quantifications

Spheres were initially removed from -20°C and allowed to reach room temperature . Following this, the spheres were fixed for 15 min at room temperature using 4% paraformaldehyde (PFA) diluted in warm 1X phosphate-buffered saline (PBS). After fixation, the slides containing the spheres were washed three times with PBS, each wash lasting 5 min. Next, the spheres were permeabilized using 1% Triton X-100 in PBS. To block nonspecific binding, the spheres were incubated for one hour in a blocking buffer composed of 0.5% bovine serum albumin (BSA) in PBS. The primary antibodies (Table S6) were prepared in blocking buffer and incubated with the spheres overnight at 4°C. Following the primary antibody incubation, the slides were rinsed three times with PBS, each rinse lasting 5 min. The secondary antibody and DAPI were added to the secondary antibody solution at a 1:400 and 1:1000 dilution, respectively, and incubated with the slides for 2 hours at room temperature in the dark. After incubation, the slides were rinsed three times with PBS, with each rinse lasting 5 min. Following the rinsing, the slides were mounted using Mowiol mounting medium and allowed to solidify overnight. For immunofluorescence signal quantification, mean intensity of E-cadherin, Vim, Krt14 and Krt18 was normalized to the tumorsphere section area (quantification were performed using Fiji v1.54m) and graphed using Prism v8.0.1.

Table S7: RNA sequencing (RNA-seq) coverage and quality statistics

Sample ID	Total number Of sequenced reads	Total number of uniquely mapped reads (Mus_musculus. GRCm38)	RNA integrity number (RIN)	Ratio of exon-mapped reads to total uniquely mapped reads (Expression Profile Efficiency)	Total number of Detected transcripts with reads ≥1
CTRL_2470	16791349	12386736	8-10	73.77%	22128
CTRL_2478	16018223	11623401	8-10	72.56%	22538
Rad_2488	17711747	13061258	8-10	73.74%	19247
Rad_2481	17084283	12889606	8-10	75.45%	23292
Rad_2492	20404002	14556325	8-10	71.34%	22677

Table S8: spatial RNA sequencing (spRNA-seq) coverage and quality statistics

Sample ID	Total number of sequenced reads	Total number of uniquely mapped reads (Mus_musculus. GRCm38)	Total number of called spots (under tissue)	Median number (and range) of uniquely mapped reads per called spot	Median number (and range) of detected genes per called spot
control	179996887	167217108	4992 (2851)	13220 (135-62362)	4266.5 (112-7959)
radiation	228343323	210075857	4992 (2398)	19502.5 (19-62862)	5170 (18-8007)

Supplementary Figures

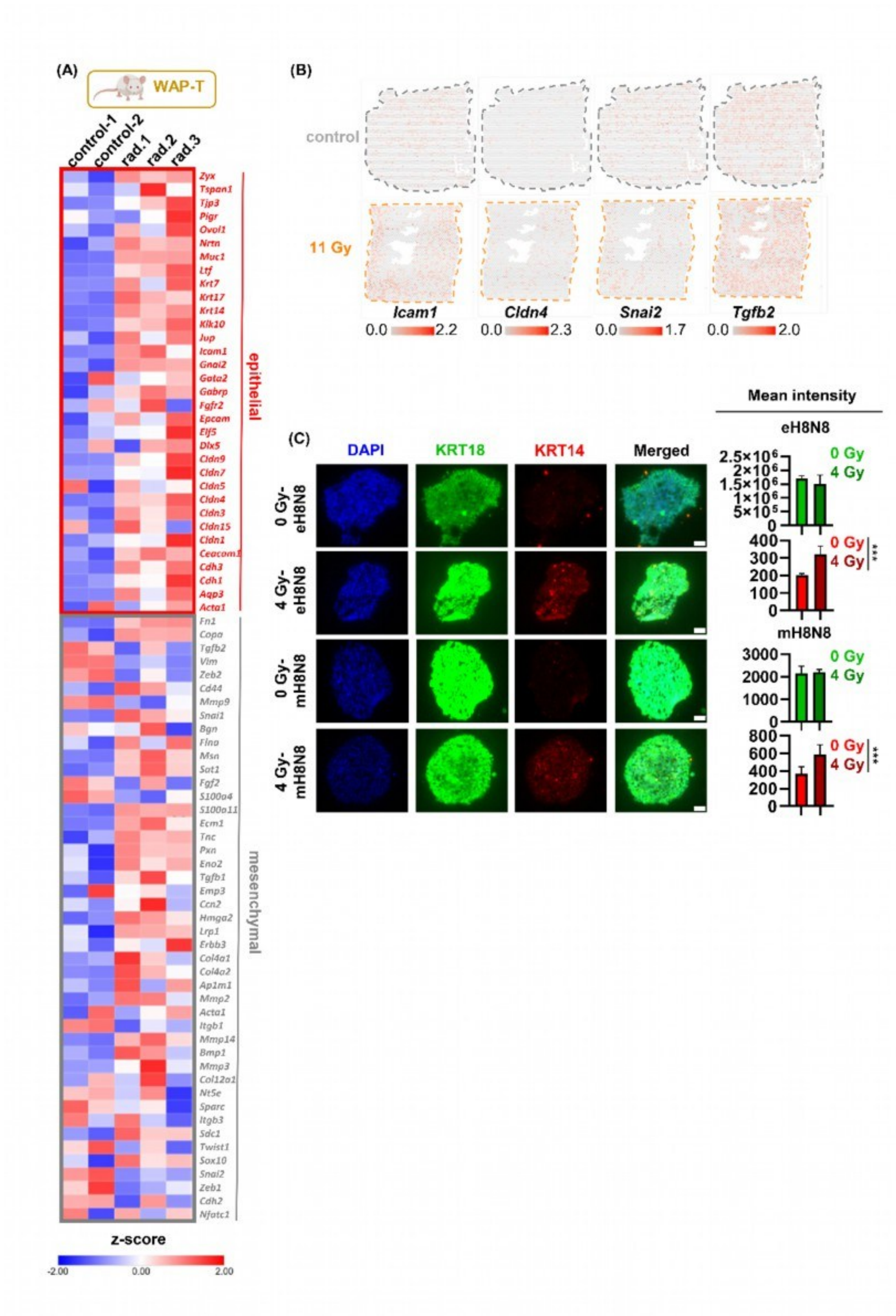

Figure S1 (A) Gene expression heatmap of epithelial and mesenchymal genes in control and RT-treated WAP-T tumors. (B) Spatial transcriptomic expression of *Icam1*, *Cldn4*, *Snai2* and *Tgfb2* in control and RT-treated WAP-T tumors. (C) Immunofluorescence staining of KRT18 and KRT14 in control and 11 Gy-treated WAP-T tumors (left panel) and their respective quantification (right panel). DAPI is used as counterstain. White scale bar in C: 200 µm. Statistics: C Student t-test, ***p≤0.001. All experiments were performed in biological triplicates.

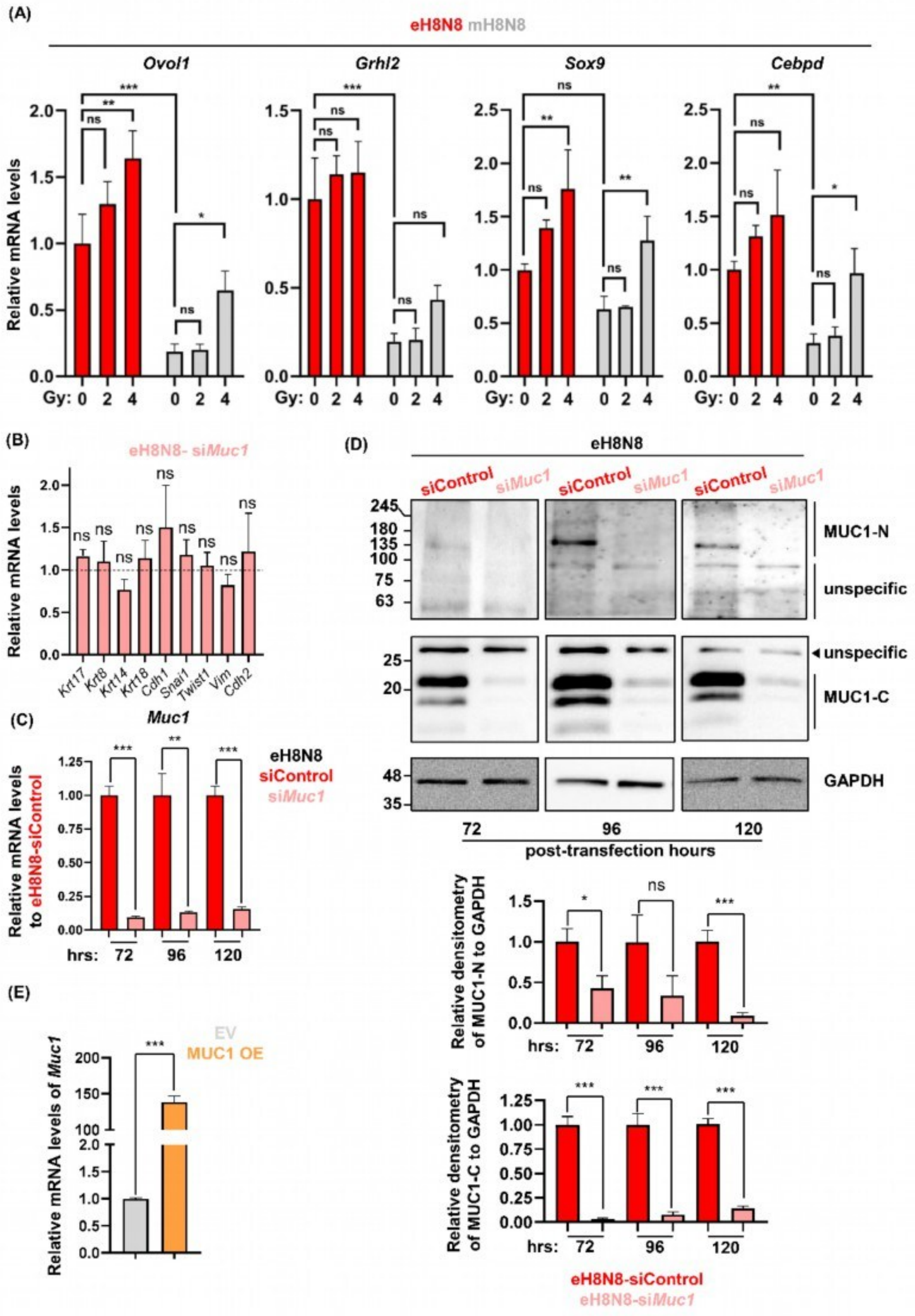

Figure S2 (A) RT-qPCR of *Ovol1*, *Grhl2*, *Sox9*, *Cebpd*, in control, 2 Gy and 4 Gy-treated eH8N8 and mH8N8 cells. (B) RT-qPCR of several epithelial and mesenchymal markers in siControl and si*Muc1*-treated eH8N8 cells. siControl-derived expression of the genes is depicted as a relative mean demarcated by the dashed line. (C) RT-qPCR of *Muc1* in siControl and siMUC1-treated eH8N8 cells at 72, 96 and 120 hours post-transfection. (D) Western blotting of MUC1-C and MUC1-N and their respective internal loading controls from one representative biological replicate in siControl- and siMUC1-treated eH8N8 cells at 72, 96 and 120 hours post-transfection (upper panel), as well as the correspnding densitometry analyses (lower panel). (E) RT-qPCR of *Muc1* in EV and MUC1 OE mH8N8 cells. Statistics: A-E Student t-test; ns: not significant, *p<0.05, **p≤0.01, ***p≤0.001. All experiments were performed in biological triplicates.
